# Tumor‐Infiltrating mregDCs Restrain Anti‐Tumor Immunity in Early Relapse HCC

**DOI:** 10.1002/advs.75695

**Published:** 2026-05-14

**Authors:** Zefan Zhang, Lin Ding, Yu Zhong, Haokang Feng, Linglong Huang, Waidong Huang, Chunqing Wang, Arne Östman, Beili Wang, Jian Zhou, Jia Fan, Wei Guo, Liang Wu, Yunfan Sun

**Affiliations:** ^1^ Department of Hepatobiliary Surgery and Liver Transplantation Liver Cancer Institute Zhongshan Hospital Fudan University Key Laboratory of Carcinogenesis and Cancer Invasion Ministry of Education Shanghai China; ^2^ Zhongshan‐BGI Precision Medical Center Zhongshan Hospital Fudan University Shanghai China; ^3^ Department of Laboratory Medicine Zhongshan Hospital Fudan University Shanghai China; ^4^ BGI Research Chongqing China; ^5^ Department of Pathology College of Basic Medicine Chongqing Medical University Chongqing China; ^6^ Department of Oncology‐Pathology Karolinska Institutet Stockholm Sweden; ^7^ College of Life Sciences University of Chinese Academy of Sciences Beijing China; ^8^ State Key Laboratory of Genome and Multi‐Omics Technologies BGI Research Shenzhen China

**Keywords:** CD161^+^CD8^+^ T cell, immune escape, MregDC, relapsed HCC

## Abstract

Emerging evidence highlights the tumor microenvironment's (TME) role in hepatocellular carcinoma (HCC), yet how tumor‐infiltrating myeloid cells drive relapse is unclear. Using full‐length single‐cell RNA sequencing (scRNA‐seq) on samples from primary and early‐relapse HCC patients, we identified a dendritic cell subset DC3, which in relapsed tumor exhibited features of mature DCs enriched in immunoregulatory molecules (mregDCs). Mechanistically, mregDCs recruit dysfunctional CD161^+^CD8^+^ T cells, which secrete TNF‐α, thereby activating the non‐canonical NF‐κB pathway to promote the differentiation of mature DCs into mregDCs via tumor necrosis factor receptor 2 (TNFR2). Our results from in vivo mouse models demonstrated that dual blockade of TNFR2 and PD‐L1 reduced tumor burden more effectively than anti‐PD‐L1 monotherapy in mregDC‐rich HCC. We also found strong interactions between mregDCs and FCN1+ monocytes, a myeloid‐derived suppressor cell (MDSC)‐like population. Our study characterizes an mregDC‐mediated immunosuppressive network in relapse HCC, nominating TNFR2 as a therapeutic target for myeloid‐focused HCC immunotherapy.

## Graphical abstract

Tumor‐infiltrating mregDCs are regulated by the TNFR2‐non‐canonical NF‐κB axis, which is mediated by TNF‐α secreted by CD161^+^CD8^+^ T cells. These mregDCs recruit CD161^+^CD8^+^ T cells via the CCL20‐CCR6 axis, forming a positive feedback loop that enhances immunosuppression, promotes early recurrence of HCC, and further confirms TNFR2 as a key therapeutic target.

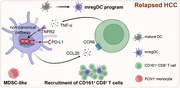

## 1 Introduction

Hepatocellular carcinoma (HCC) represents a formidable global health challenge, currently ranking as the third most common cause of cancer‐related deaths worldwide [1]. Although surgical interventions, including hepatic resection and liver transplantation, remain the cornerstone of potentially curative treatment [2, 3], their therapeutic potential is substantially undermined by high recurrence rates approaching 70% within five years post‐resection [4]. This persistent clinical challenge not only complicates disease management but also contributes to disappointing long‐term survival outcomes.

The advent of immunotherapy, particularly immune checkpoint inhibitors, has revolutionized HCC treatment paradigms [5, 6]. However, current strategies primarily focus on modulating T‐cell‐mediated immunity, demonstrating clinical efficacy in only a limited patient subset [7]. These therapeutic limitations underscore the critical need for a more comprehensive understanding of the tumor immune microenvironment and the exploration of alternative immune cell targets for next‐generation HCC immunotherapies [8].

As the most potent professional antigen‐presenting cells (APCs), dendritic cells (DCs) serve as master regulators of both anti‐tumor immunity and immune tolerance [9]. The DC compartment comprises two major lineages: plasmacytoid DCs (pDCs) and conventional DCs (cDCs), with the latter further classified into three functionally distinct subsets (cDC1, cDC2, and DC3) based on their phenotypic and functional specialization [10, 11].

The cDC1 subset has been strongly associated with favorable clinical outcomes, primarily through its exceptional capacity to cross‐present tumor antigens via MHC class I and activate cytotoxic CD8^+^ T cell responses. In contrast, cDC2s specialize in MHC class II‐mediated antigen presentation to CD4^+^ T cells, orchestrating Th1, Th2, and Th17 polarization programs. DC3s represent a recently characterized pro‐inflammatory subset with unique functional attributes [11]. Most intriguingly, a novel DC population termed “mature regulatory DCs” (mregDCs) has emerged as a critical immunomodulatory player, characterized by the co‐expression of maturation markers and immunoregulatory molecules [12]. These dual‐functional APCs demonstrate remarkable plasticity upon tumor antigen encounter. Growing evidence has documented their presence across various malignancies, including triple‐negative breast cancer and non‐small cell lung carcinoma [12, 13, 14].

In the HCC context, recent studies have revealed DC accumulation in recurrent tumors [13, 15]. However, the complex bidirectional communication between DC subsets and the evolving tumor microenvironment remains poorly understood. A systematic investigation into DC subset heterogeneity, their functional adaptation, and their dynamic cellular crosstalk within the HCC ecosystem is therefore imperative for developing novel, precision immunotherapy approaches.

Through integrative full‐length single‐cell RNA sequencing and spatial transcriptomic analysis, we discovered a distinct dendritic cell population exhibiting the mregDC signature in early recurrent HCC. Our investigation revealed that these mregDCs engage in a reciprocal interaction with innate‐like CD161^+^CD8^+^ T cells—a lymphocyte subset we previously identified as being preferentially expanded in relapsed HCC [15]. Mechanistically, these CD161^+^CD8^+^ T cells secrete tumor necrosis factor‐α (TNF‐α) to promote mregDC differentiation, while mregDCs in turn recruit additional CD161^+^CD8^+^ T cells through CCL20‐CCR6‐mediated chemotaxis, thereby establishing a self‐reinforcing immunosuppressive loop within the relapsed tumor niche. To therapeutically target this pathogenic circuit, we demonstrated that TNFR2 blockade effectively disrupts mregDC‐mediated immunosuppression. Notably, combining TNFR2 inhibition with immune checkpoint blockade resulted in synergistic antitumor activity, significantly enhancing treatment efficacy compared to monotherapy approaches. Our study makes three key contributions: (1) clinical validation of mregDCs as critical orchestrators of the immunosuppressive tumor microenvironment in recurrent HCC; (2) mechanistic demonstration that TNFR2 signaling drives mregDC differentiation; and (3) preclinical proof‐of‐concept for TNFR2 blockade as a novel therapeutic strategy. These findings provide a strong rationale for developing combination immunotherapies targeting this pathway to achieve durable tumor control in recurrent HCC, with TNFR2 inhibition emerging as a particularly promising therapeutic approach.

## 2 Result

### 2.1 Single‐Cell Profiling of Primary and Early‐Relapse HCC Reveals Divergent Prognostic Roles of DC3s

**FIGURE 1:**
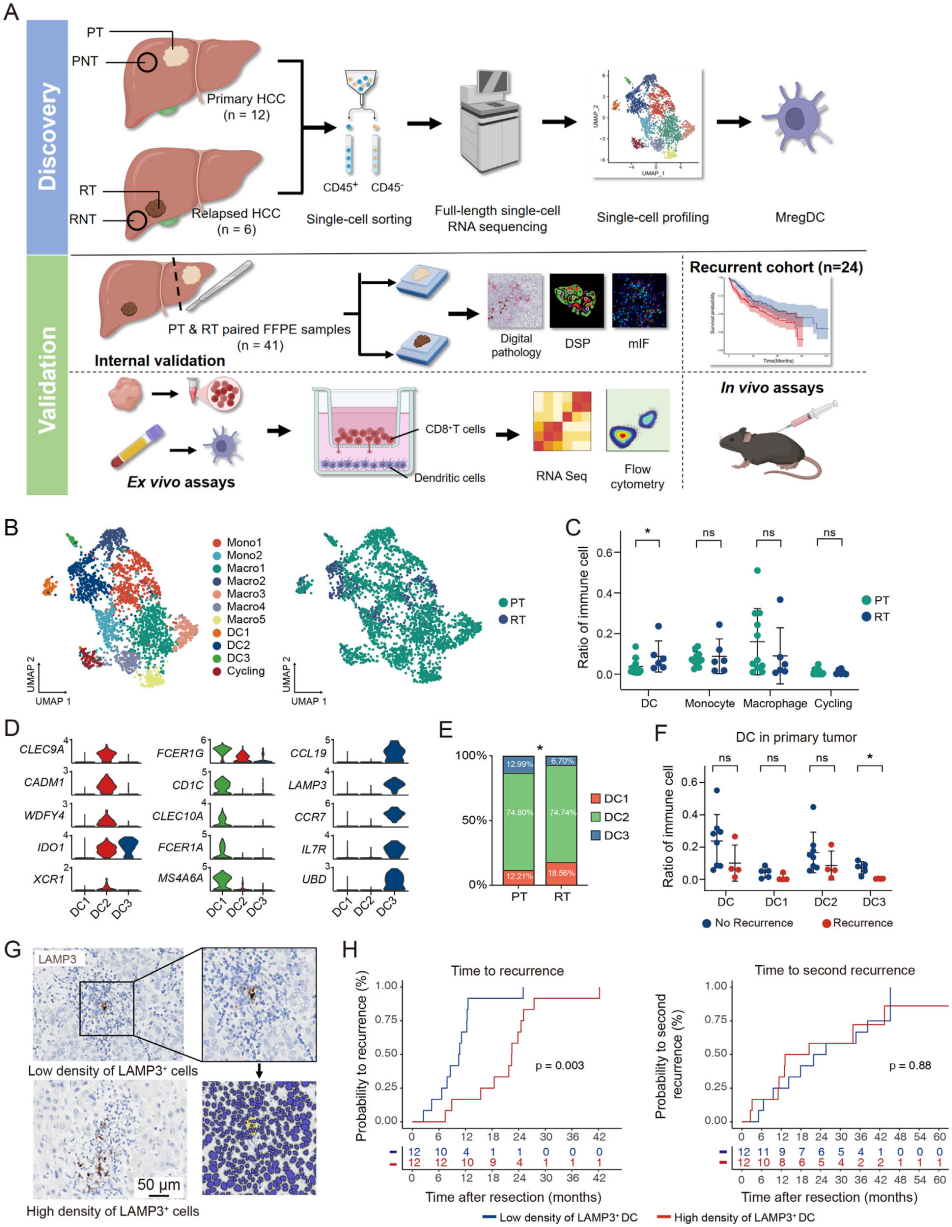
Single‐cell profiling of primary and early‐relapse HCC elucidates the existence of a distinct subset of dendritic cells that exhibits a strong association with poor prognosis. (A) Schematic overview of study design and analytical pipeline. Upper panel: Discovery cohort and single‐cell transcriptomic analysis. Lower panel: Validation cohort and experimental corroboration. (B) UMAP plots provide a visual representation of the clustering of myeloid cells from 18 patients based on their cell types (left panel) and the source of each cell (right panel). (C) Scatterplots illustrating the fraction of myeloid cells subtypes in PTs and RTs, respectively (12 patients in PT group, 6 patients in RT group). (D) Violin plot depicting the expression of marker genes for the three identified DC subsets. (E) Scatterplots showing the distinct ratio of DCs in recurrent or non‐recurrent group after resection of PT. (F) The stacked bar graphs visually depict the distribution of DC subsets in tumor versus adjacent tumor samples, as well as in RTs versus PTs. Different subtypes of DC labeled by distinct colors (8 patients in no recurrence group, 4 patients in recurrence group). (G) Representative images of IHC staining of LAMP3^+^ cells (DC3s) in Formalin‐fixed paraffin‐embedded (FFPE) tissues, illustrating the presence of LAMP3^+^ cells (DC3s) at low or high densities by HALO. Scale bar, 50 µm. (H) Kaplan‐Meier plot of probability to recurrence after resection in our validation cohort, comparing low versus high density of LAMP3^+^ DC3 in patients with PTs (left panel) and RTs (right panel). Besides, the log‐rank test results are indicated in the plot (*n* = 24 patients).

Building upon our previous single‐cell transcriptomic study of early‐relapse HCC [15], which primarily characterized tumor‐infiltrating T cell populations in relapsed tumors (RT), we now extend our investigation to comprehensively profile the myeloid compartment, a critical yet understudied component of the tumor immune microenvironment. Using the same discovery cohort from our prior study [15], consisting of scRNA‐seq data from 6 genomically confirmed metastatic RT samples, which recurred within two years after curative resection without systemic therapy, and 12 treatment‐naive primary tumors (PT), we performed an in‐depth analysis of myeloid cell populations. For validation, we utilized paired primary‐relapse FFPE specimens from 41 HCC patients in our original validation cohort (Figure 1A). After applying stringent quality control filters and excluding non‐myeloid lineages, we retained 2,948 high‐quality myeloid cells for downstream analysis. Unsupervised clustering revealed 11 transcriptionally distinct myeloid subsets, including 2 monocyte clusters, 5 macrophage clusters, 3 dendritic cell clusters, and 1 cluster of cycling cells, each characterized by unique gene expression signatures (Figure 1B; Figure S1B). The composition of these myeloid cells varied significantly between sample sources (Figure 1B) and between tumor and normal tissues (Figure S1A). Most notably, we observed a marked enrichment of DCs in early‐relapse HCC compared to primary tumors (Figure 1C), with their proportion increased within the myeloid compartment, suggesting a potential role for DCs in mediating tumor recurrence. This finding prompted us to further investigate the functional contribution of DC subsets to HCC relapse.

To systematically characterize DC heterogeneity, we classified all DCs into three distinct subsets based on established marker genes: cDC1 (CLEC9A^+^CADM1^+^), cDC2 (CLEC10A^+^CD1C^+^), and DC3 (CCL19^+^LAMP3^+^CCR7^+^IL7R^+^CCL22^+^) (Figure 1D; Figure S1C). Comparative analysis revealed that DC3s were predominantly localized within tumor tissues, with their relative proportion being significantly higher in PTs compared to RTs (Figure 1E). Notably, in primary HCC specimens, patients who subsequently developed recurrence exhibited markedly reduced DC3 proportions compared to non‐recurrent cases (Figure 1F; Figure S1D). However, this association was not observed in RTs, where DC3 levels showed no correlation with secondary recurrence risk. The immunohistochemistry assay was performed on FFPE samples from 24 HCC patients with follow‐up information after resection of recurrent HCC, and patients were stratified into two groups based on the median value of immunohistochemical staining results. We yielded intriguing clinical correlations: High LAMP3^+^ DCs infiltration in PTs was associated with improved recurrence‐free survival (Figure 1G,H), suggesting a potential protective role against early recurrence. In striking contrast, DC3 abundance in RTs showed no significant prognostic value, indicating a possible functional alteration or context‐dependent role of these cells in the recurrent tumor microenvironment.

### 2.2 Characterization of DC3 Subsets in RT Revealing the Molecular Features of mregDC

**FIGURE 2:**
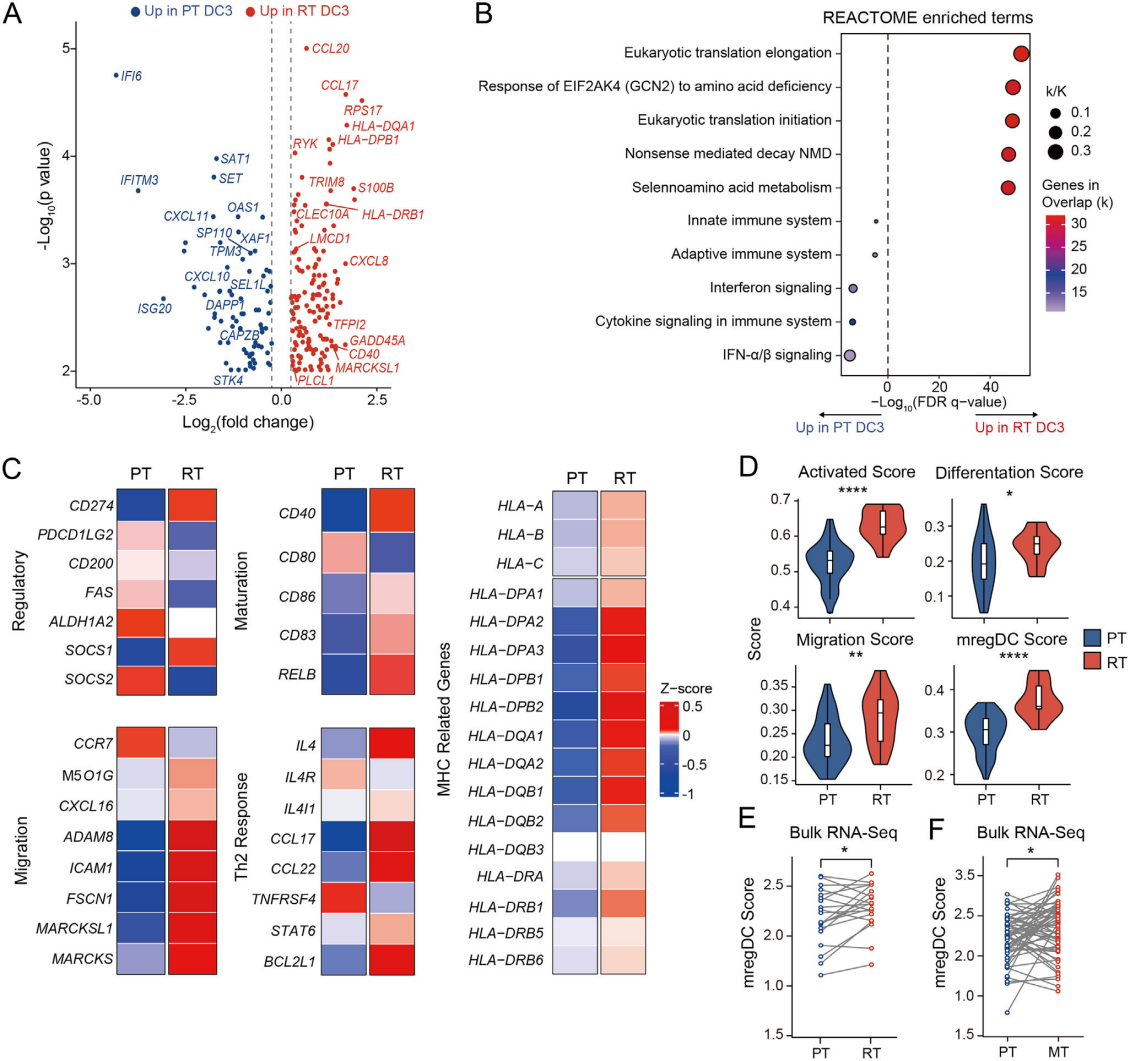
The transcriptome atlas of DC3s was similar to that of mregDCs. (A) Volcano plot showing differentially expressed genes between DC3 in PTs and those in RTs. The names of the most significant genes were indicated in the plots. (B) Dot plot showing the enriched activated pathways in DC3 cells in RTs and PTs. (C) Heatmaps showing the Z‐score of specific genes in DC3 between PT and RT, including genes associated with immunoregulation, maturation, migration, Th2 response, and antigen presentation. (D) Violin plot indicating the differences in the activation score, differentiation score, migration score and mregDC score between RTs and PTs, respectively. (E) Bar plot showing the comparison of mregDC scores between paired PT and RT based on bulk RNA‐seq data from the validation cohort (*n* = 41 paired PT and RT). (F) Bar plot showing the comparison of mregDC scores between paired primary HCC and extrahepatic metastasis (*n* = 78 paired PT and RT).

Our comparative transcriptomic analysis revealed markedly distinct transcriptional profiles in DC3 cells between PT and RT. Strikingly, RT DC3s exhibited pronounced upregulation of key chemokine genes (CCL20 and CCL17; Figure 2A), suggesting enhanced migratory potential and pro‐inflammatory activity within the recurrent tumor niche. In contrast, PT DC3s displayed a predominant type I interferon signature, characterized by significant induction of interferon‐stimulated genes including IFITM3 and IFI6 [16], indicative of robust antiviral defenses and heightened antitumor immunity. REACTOME pathway analysis uncovered fundamental mechanistic differences underlying these phenotypic distinctions (Figure 2B). RT DC3s showed significant enrichment in protein biosynthesis pathways, particularly eukaryotic translation elongation, suggesting a metabolic reprogramming toward cellular maintenance and homeostasis. Conversely, PT DC3s demonstrated comprehensive activation of immune effector pathways, encompassing both innate and adaptive immune responses, with particularly strong engagement of type I interferon signaling cascades. These findings collectively suggest a dynamic functional evolution of DC3 cells during tumor progression, transitioning from an immunologically active state in primary tumors to a more metabolically adapted phenotype in recurrent disease.

Deep phenotyping revealed that RT DC3s exhibit elevated expression of maturation markers, migration‐associated receptors, costimulatory molecules, TH2‐polarizing factors, and antigen‐presentation machinery, including the immunoregulatory molecule PD‐L1 (*CD274*)—a profile closely resembling mature regulatory DCs (mregDCs) (Figure 2C) [12]. To systematically assess this resemblance, we performed single‐sample gene set enrichment analysis (ssGSEA) of an established mregDC signature across all myeloid subsets. Strikingly, only DC3s displayed high enrichment scores, suggesting that the mregDC phenotype is predominantly restricted to this subset (Figure S2A). Further analysis confirmed that RT DC3s exhibited enhanced activation, migratory capacity, differentiation potential, and mregDC‐associated signatures compared to their PT counterparts (Figure 2D) [11, 17]. To validate these findings, we performed gene set enrichment analysis (GSEA) on RNA‐seq data from an independent paired PT/RT cohort (n = 41 pairs), which confirmed that the mregDC expression score was significantly elevated in RT DC3s, indicating that mregDCs are more abundant in relapse lesions (Figure 2E). Intriguingly, when we extended this analysis to a separate cohort of 78 paired primary HCC and extrahepatic metastatic tumors (MTs) from our prior study [18], we observed a similar accumulation of mregDC‐like DC3s in metastatic lesions (Figure 2F). This finding suggests a potential link between mregDC polarization and metastasis, possibly through facilitating immune evasion or priming the pre‐metastatic niche [19].

Taken together, our results reveal that DC3s undergo profound functional reprogramming in recurrent tumors, adopting an mregDC‐like phenotype marked by enhanced activation and differentiation programs but compromised immunostimulatory capacity. The significant enrichment of these regulatory DC3s in both recurrent and metastatic lesions strongly implicates their pivotal role in shaping an immunosuppressive microenvironment that fosters tumor progression.

### 2.3 Spatial Profiling of mregDC Distribution in the Tumor Microenvironment of Early‐Relapse HCC

**FIGURE 3:**
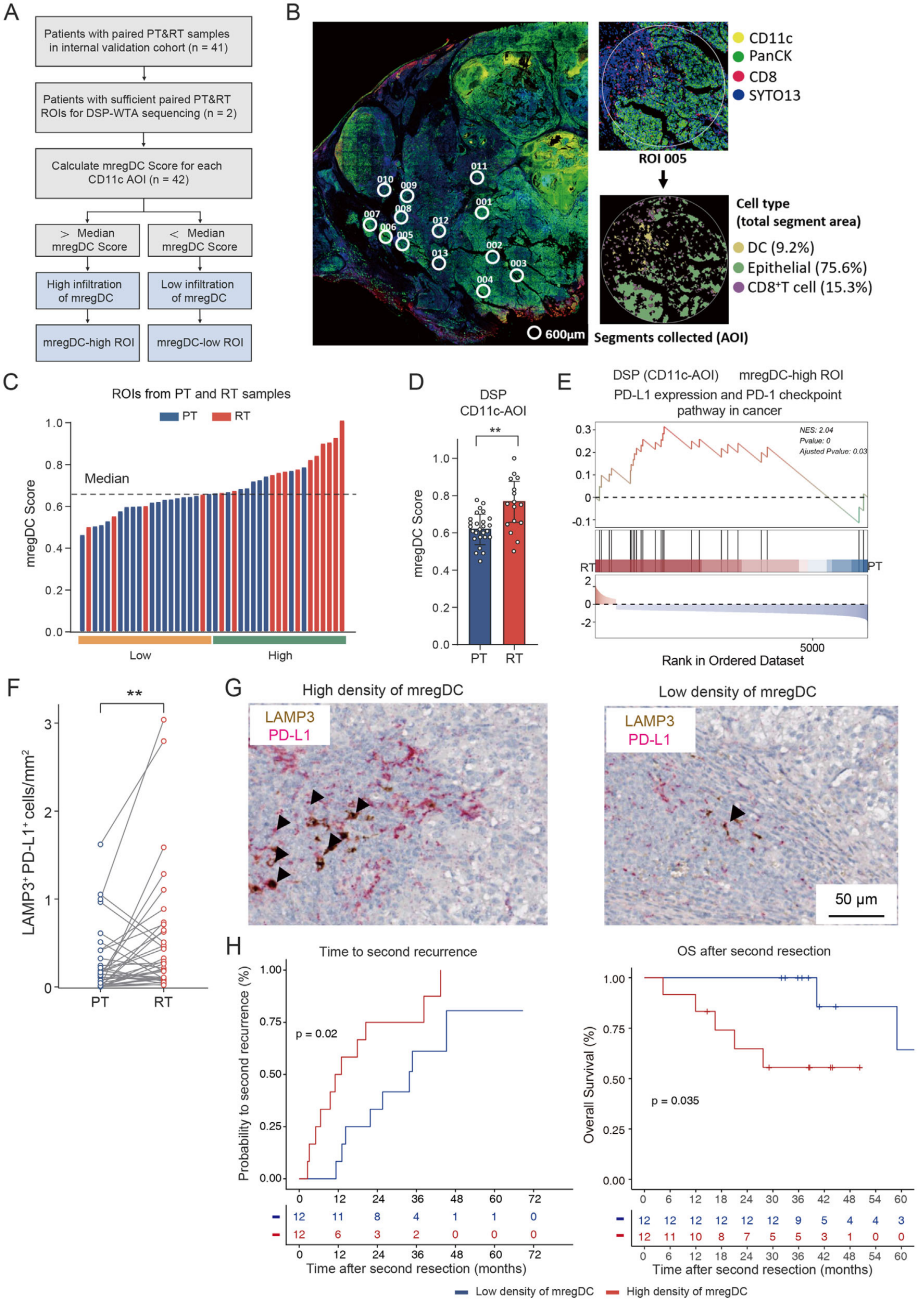
Spatial transcriptome analysis of mregDCs by DSP. (A) The grouping strategy of ROIs was shown in the flow chart. (B) Whole transcriptome digital spatial profiling. Immunofluorescence image of consecutive sections from the tumor FFPE block, showing selected regions of interest (left panel). WTA DSP was performed on 2 HCC patients with RTs and PTs. The representative ROI with segmentation masks used to enrich the epithelial cells, DCs and CD8^+^ T cells, and percent of total segment area occupied by each compartment. (C) Box plot illustrating the mregDC scores in all CD11c AOIs, and the corresponding ROIs were divided into high or low mregDC groups using the median value of mregDC score as the cutoff (*n* = 42). (D) Box plot illustrating the comparison of mregDC scores in CD11c AOIs between PTs and RTs in DSP (*n* = 26 in PT group, *n* = 16 in RT group). (E) GSEA analysis showing the enrichment of gene sets associated with PD‐L1 expression and PD‐1 checkpoint pathway in CD11c AOIs with high mregDC scores from RT samples compared to those from PT samples. (F) Dot plots representing the density of LAMP3^+^PD‐L1^+^ cells (*n* = 41 paired PT and RT). (G) Representative images of double immunohistochemical staining of LAMP3^+^PD‐L1^+^ cells in RT (upper panel) and PT (lower panel). The scale bar represents 50 µm. (H) Kaplan‐Meier curves for overall survival and probability to second recurrence. Patients were divided into high and low density of mregDC groups using the median value as the cutoff. *P* values were calculated using the log‐rank test (*n* = 24).

To delineate the spatial distribution of mregDCs within the tumor microenvironment (TME), we employed GeoMx Digital Spatial Profiler Whole Transcriptome Atlas (DSP‐WTA) analysis. From our cohort of paired PT and RT samples, we selected two representative cases with sufficient regions of interest (ROIs) for spatial transcriptomic profiling. A total of 42 manually annotated areas of interest (AOIs) were analyzed following multiplex immunofluorescence staining for CD11c (dendritic cells), PanCK (epithelium), CD8 (cytotoxic T cells), and SYTO83 (nuclear counterstain) (Figure 3A,B). CD11c‐enriched AOIs were subsequently subjected to DSP‐WTA sequencing to characterize dendritic cell‐specific transcriptional programs. Unlike prior DSP studies that often restrict AOIs to predefined immune “hotspots,” our sampling was performed in a representative and balanced manner across intratumoral, invasive margin, and stromal compartments. Importantly, the same unbiased strategy was applied to both PT and RT cohorts, ensuring comparable AOI composition between groups. This approach not only minimizes histological selection bias but also enhances the robustness of biological interpretation, allowing observed differences in immune programs to be confidently attributed to relapse status rather than sampling artifacts.

We quantified mregDC infiltration by calculating transcriptomic mregDC signature scores for each CD11c‐enriched AOI, classifying ROIs as mregDC‐high (above mean score) or mregDC‐low (below mean). RT‐derived AOIs exhibiting elevated mregDC scores compared to PT samples (Figure 3C,D). These findings spatially validate our prior observation that DC3s preferentially adopt an mregDC phenotype in recurrent tumors.

Given prior reports implicating PD‐L1^+^LAMP3^+^ DCs as canonical mregDCs [12, 20], we assessed their spatial distribution. Consistent with our bulk RNA‐seq data (Figure 2C), the result of GSEA revealed significant enrichment of PD‐L1 expression and PD‐1 checkpoint pathways in RT versus PT AOIs (Figure 3E). Immunofluorescence validation confirmed a higher frequency of LAMP3^+^PD‐L1^+^ DCs in RTs (Figure 3F,G), with elevated densities correlating with shorter overall survival and relapse‐free intervals (Figure 3H), suggesting their clinical relevance.

Extending our findings beyond HCC, we applied the mregDC signature to TCGA pan‐cancer data. Mirroring previous reports [21], mregDCs were ubiquitously detected across malignancies. Notably, high mregDC scores predicted worse prognosis in most cancer types by both log‐rank testing and Cox regression (Figure S3A,B), underscoring their conserved role in fostering immunosuppressive TMEs.

Our integrated spatial and transcriptomic analyses reveal recurrent tumors as privileged niches for mregDC accumulation, where these cells likely drive immune tolerance. The association between mregDC abundance and adverse outcomes across cancers positions them as potential biomarkers and therapeutic targets in tumor immunity.

### 2.4 CD161^+^CD8^+^ T Cell‐Derived TNF‐α Induced Differentiation of Mature DCs into mregDCs

**FIGURE 4:**
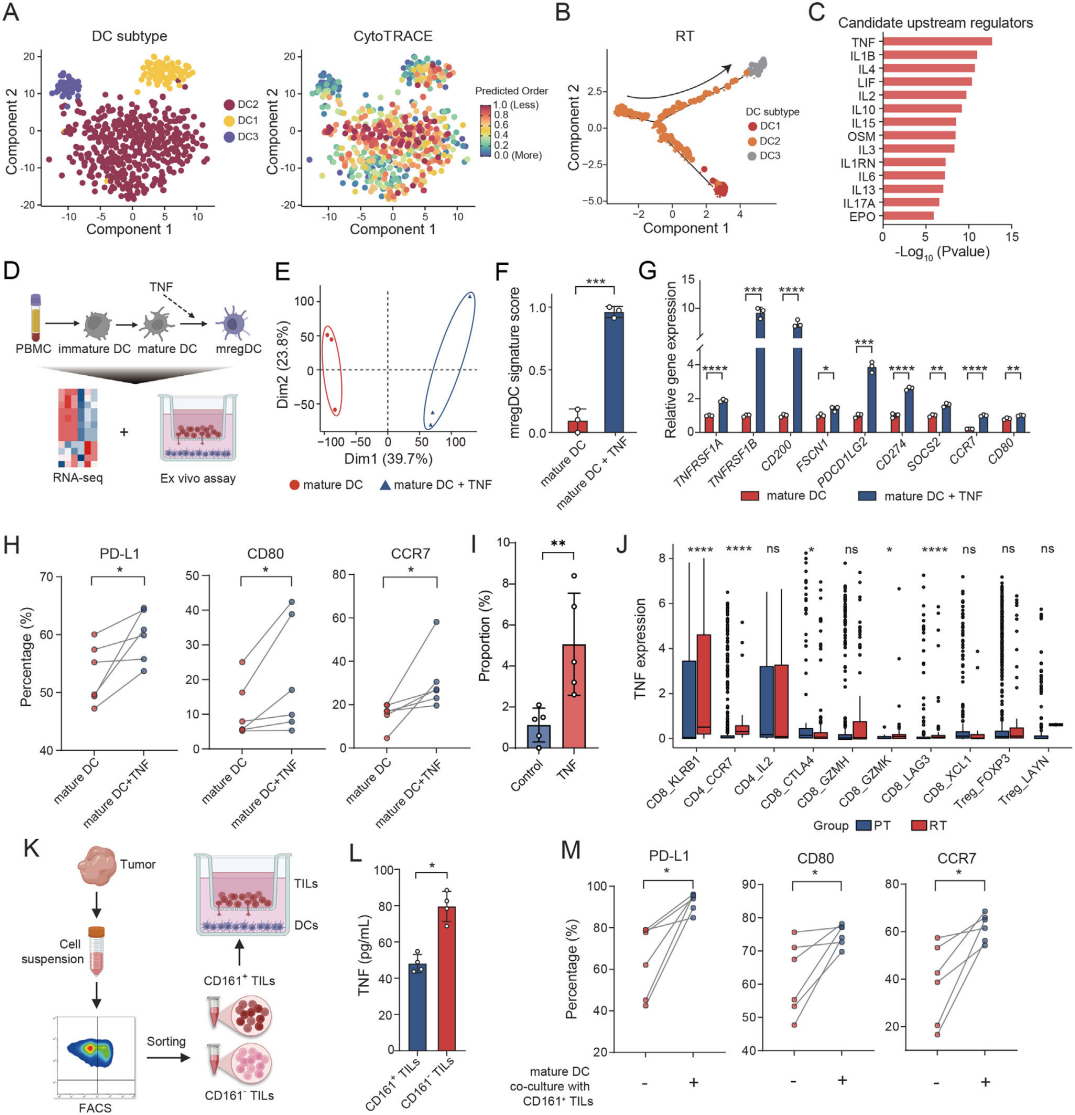
mregDC program is mediated by TNF‐α. (A, B) Analysis of mregDC differentiation trajectory. CytoTRACE analysis showing DC3 (mregDC) represents a terminal differentiation state in RT (A), Monocle2‐based developmental trajectory analysis revealing DC3 potentially differentiated from DC2 in RT (B). (C) Box plot illustrating candidate upstream regulators by IPA analysis. (D) Schematic illustration of the investigation. (E) PCA plots showing transcriptional profiles of TNF‐α‐treated versus untreated mature DCs (*n* = 3 per group). (F) Box plot illustrating the comparison of mregDC scores between mature DCs and mature DCs treated with TNF‐α (*n* = 3 per group). (G) Box plot showing the expression of key mregDC signature genes in TNF‐α‐treated versus untreated mature DCs, as measured by qRT‐PCR (*n* = 3 per group). (H) Dot plots comparing PD‐L1, CD80, CCR7 expression between paired mature DCs and mature DCs treated with TNF‐α (*n* = 6 per group). (I) Box plot illustrating proportion of tumor‐infiltrating mregDCs among total immune cells in tumor tissues under different treatment conditions (*n* = 5 per group). (J) Box plot illustrating the *TNF* expression of different T cell subsets in patients with RT and PT. (K) Schematic representation of the *ex vivo* co‐culture experimental strategy. (L) Box plot illustrating the expression level of TNF‐α in conditioned medium of CD161^+^CD8^+^ T cells and CD161^−^ CD8^+^ T cells determined by ELISA (*n* = 4 per group). (M) Dot plots comparing PD‐L1, CD80, CCR7 expression between mature DCs and mature DCs co‐culture with CD161^+^CD8^+^ T cells (*n* = 6 per group).

Previous studies have established that mregDCs arise from immature DCs through tumor microenvironment‐derived signals [22]. In our RT samples with prominent mregDC enrichment, CytoTRACE and Monocle2 analyses positioned mregDCs at the terminal end of the differentiation trajectory, exhibiting maximal maturation states (Figure 4A,B; Figure S4A,B). To identify upstream regulators of this transition, we performed ingenuity pathway analysis (IPA), which revealed TNF as a top candidate driver of mregDC development (Figure 4C).

To experimentally validate these predictions, we first generated fully mature DCs through ex vivo differentiation of human peripheral blood mononuclear cells (PBMCs). These mature DCs were then exposed to TNF‐α treatment (Figure 4D; Figure S4C), given this cytokine's well‐documented pleiotropic roles in immune regulation. Comprehensive transcriptomic analysis conducted after 48 h of treatment demonstrated clear segregation between TNF‐α‐treated and untreated DC populations by principal component analysis (PCA) (Figure 4E; Figure S4D), indicative of substantial global transcriptional reprogramming. Quantitative assessment revealed that TNF‐α‐treated DCs displayed significantly elevated mregDC signature scores compared to untreated controls (Figure 4F). Furthermore, we observed marked upregulation of genes associated with canonical mregDC features, including immunoregulatory functions (*CD274*), maturation markers (*CD80*), migratory capacity (*CCR7*), as well as the cytokine's cognate receptors (*TNFRSF1A* and *TNFRSF1B*) (Figure 4G).

To complement these transcriptional findings at the protein level, we performed multiparameter flow cytometry analysis. Our results demonstrated that TNF‐α stimulation significantly enhanced the expression of multiple immunoregulatory molecules in mature DCs, including the inhibitory ligand PD‐L1, the costimulatory molecule CD80, and the migration‐associated receptor CCR7 (Figure 4H). Importantly, control experiments comparing immature DCs, mature DCs, and TNF‐α‐treated immature DCs established the specificity of this effect, with only TNF‐α‐exposed mature DCs acquiring the full mregDC phenotype (Figure S4E,F). To delineate the pivotal biological function of TNF‐α in driving the mregDC phenotype, we established a subcutaneous tumor model in immunocompetent C57BL/6 mice using the Hepa1‐6 murine HCC cell line, and administered intratumoral injection of syngeneic mature DCs into established models. Subsequent flow cytometric analysis revealed that tumor tissues subjected to intratumoral TNF‐α administration exhibited a markedly increased infiltration of mregDCs (CD45^+^Lin^−^MHC‑II^+^CD11c^+^CD80^+^PD‑L1^+^) compared with the PBS‐treated control group (Figure 4I;Figure S4G). These results underscore TNF‐α’s critical and stage‐specific role in driving the terminal differentiation of mature DCs into mregDCs.

Having established TNF‐α’s capacity to induce mregDC differentiation, we next sought to identify its physiological source within the tumor microenvironment. Single‐cell analysis revealed that *TNF* expression was most prominently enriched within subsets of CD8_KLRB1, CD4_IL2, NK cells and myeloid cells, strikingly, *TNF* expression levels were significantly elevated in CD161^+^CD8^+^ T cells, NK cells and myeloid cells isolated from early recurrent HCC lesions, relative to those from primary HCC (Figure 4J; Figure S4H). Given the characteristic numerical predominance of CD161^+^CD8^+^ T cells infiltrating in early recurrent HCC microenvironment (Figure S4I–K), we posited that this CD8^+^ T cell subset constitutes a major cellular source of TNF‐α. This finding corroborates our previous report documenting the enrichment of innate‐like CD161^+^CD8^+^ T cells with dysfunctional cytotoxicity and low expansion in recurrent hepatocellular carcinoma, where single cell analysis and flow cytometric analysis revealed markedly reduced granzyme B (GZMB) expression together with impaired effector functions [15]. To establish a direct causal relationship, we developed an ex vivo co‐culture system (Figure 4K). Freshly isolated CD161^+^ and CD161^−^CD8^+^ T cell subsets from HCC specimens were assessed for cytokine secretion profiles. ELISA quantification of culture supernatants confirmed that CD161^+^CD8^+^ T cells secreted significantly greater quantities of TNF‐α compared to their CD161^−^ counterparts (Figure 4L). When mature DCs were co‐cultured with these distinct T cell populations, only CD161^+^CD8^+^ T cells could effectively induce the canonical mregDC phenotype, as evidenced by upregulated expression of *CD274*, *CD80*, and *CCR7* (Figure 4M; Figure S4L).

Collectively, these findings provide compelling evidence that TNF‐α secreted by tumor‐infiltrating CD161^+^CD8^+^ T cells plays a crucial role in promoting the transformation of mature DCs into mregDCs within the tumor microenvironment.

### 2.5 TNFR2‐Dependent Activation of the Non‐Canonical NF‐κB Axis Mediated Differentiation of mregDC

**FIGURE 5:**
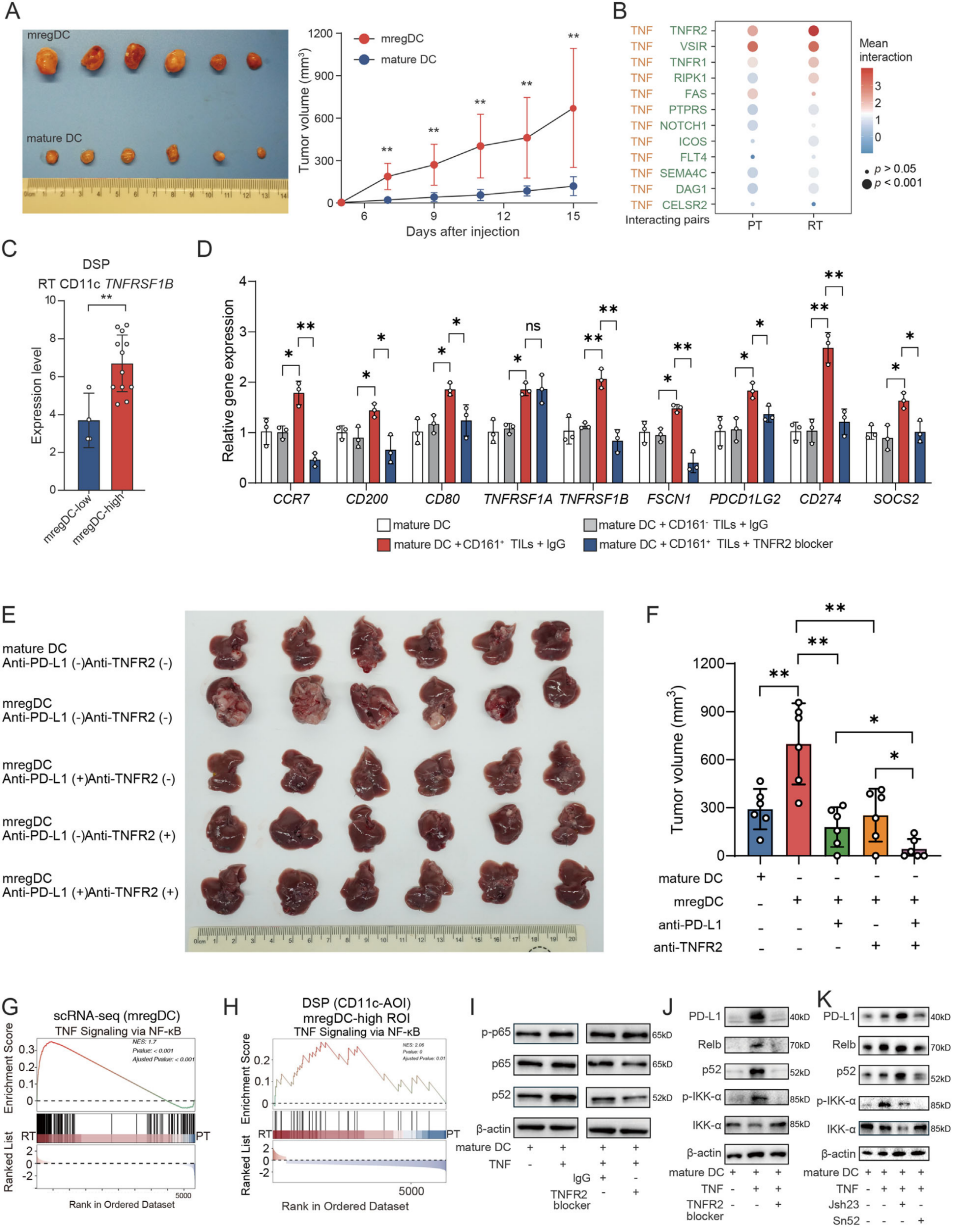
TNFR2‐mediated non‐canonical NF‐κB signaling drives mregDC development and tumor progression. (A) Tumor growth curves in mice co‐injected with Hepa1‐6 cells and either mature DCs or mregDCs (*n* = 6 per group). (B) CellphoneDB analysis showing TNF‐TNFR2 interaction scores between mregDC‐high recurrent tumors and mregDC‐low primary tumors. (C) Digital spatial profiling (DSP) analysis of *TNFRSF1B* expression in mregDC‐high versus mregDC‐low areas of interest (AOIs, *n* = 4 in mregDC‐low group, *n* = 12 in mregDC‐high group). (D) Box plot showing the expression of key mregDC signature genes in DCs under different treatment conditions, as measured by qRT‐PCR. (*n* = 3 per group). (E, F) Image of tumor‑bearing livers from mice in different treatment groups, and quantitative comparison of hepatic tumor volume among the indicated groups (*n* = 6 per group). (G) GSEA analysis showing the enrichment of gene sets associated with TNF Signaling via NF‐κB in mregDCs from RT samples compared to those from PT samples. (H) GSEA analysis showing the enrichment of gene sets associated with TNF signaling via NF‐κB in CD11c AOIs with high mregDC scores from RT samples compared to those from PT samples. (I, J) Western blot analysis of canonical and non‐canonical NF‐κB pathway components and mregDC markers following TNFR2 blockade. (K) Expression of mregDC markers following treatment with pathway‐specific inhibitors (Sn52 for non‐canonical and Jsh23 for canonical NF‐κB pathway).

To elucidate the functional impact of mregDCs on tumor progression, we established an in vivo model system by subcutaneously co‐injecting Hepa1‐6 tumor cells with either mature DCs or mregDCs into immunocompetent C57BL/6 mice. Strikingly, tumors co‐injected with mregDCs exhibited significantly accelerated growth kinetics compared to those receiving mature DCs (Figure 5A), demonstrating a direct pro‐tumorigenic role for mregDCs in tumor development.

Employing comprehensive ligand‐receptor interaction analysis (CellphoneDB), we systematically mapped intercellular communication networks in mregDC‐high versus mregDC‐low AOIs. Our analysis revealed significant enrichment of TNF‐TNFR2 (*TNFRSF1B*) interactions specifically in mregDC‐high AOIs derived from recurrent tumors (Figure 5B). Notably, among TNF receptor superfamily members, TNFR2 exhibited the highest interaction weights, suggesting its potential role in mediating TNF signaling within the tumor microenvironment (Figure S5A). Moreover, DSP analysis confirmed higher expression of TNFR2 in mregDC‐high AOIs (Figure 5C). In contrast, while TNFR1 (*TNFRSF1A*) displayed increased interaction weights in recurrent tumors, its expression pattern lacked correlation with mregDC abundance (Figure S5B), underscoring the specificity of the TNFR2 pathway in mregDC regulation.

Having identified CD161^+^CD8^+^ T cells as the primary TNF‐α source, we further investigated the TNF‐TNFR2 axis in CD161^+^CD8^+^ T cell‐driven mregDC differentiation. In co‐culture experiments with mature DCs, CD161^+^CD8^+^ T cells, but not CD161^−^ TILs, robustly upregulated both mregDC signature genes and TNF pathway components (Figure 5D). This effect was markedly attenuated by TNFR2 blockade, which suppressed mregDC marker expression (Figure 5D), confirming TNFR2 as the principal mediator of this crosstalk. We further treated recombinant TNF‑α‑induced mregDCs with TNFR2 inhibitor and obtained consistent results (Figure S5C), validating the essential regulatory function of TNFR2.

Given TNFR2's critical role in mregDC development and the clinical limitations of PD‐L1 monotherapy [23, 24, 25], we investigated whether TNFR2 blockade could potentiate PD‐L1 inhibition. In mice receiving mature DCs and tumor cells, adding TNFR2 blocker to PD‐L1 antibody treatment showed no significant improvement in tumor control (Figure S5D). In contrast, when tumors were established with mregDCs, the combination therapy demonstrated superior anti‐tumor efficacy compared to PD‐L1 antibody alone (Figure S5E), highlighting the therapeutic potential of targeting TNFR2 specifically in mregDC‐rich tumors. We further established a murine model featuring orthotopic liver implantation of subcutaneous tumor grafts, and administered intratumoral injection of either mature DCs or mregDCs into hepatic tumors, coupled with distinct therapeutic regimens. By assessing the formation and progression of orthotopic liver tumors, we further validated that intratumoral mregDCs, in comparison with mature DCs, potently promoted tumor growth. Moreover, in the setting of substantial mregDC infiltration within the tumor microenvironment, combined administration of anti‐PD‐L1 antibody and anti‐TNFR2 antibody markedly suppressed tumor growth, as compared with either monotherapy alone or the untreated control group (Figure 5E,F). Additionally, mregDCs were isolated from harvested murine tumor tissues, and the expression levels of mregDC signature genes across distinct experimental groups were subsequently determined by PCR analysis. The results demonstrated that treatment with anti‐TNFR2 antibody significantly blunted the characteristic upregulation of mregDC‐related signature genes (Figure S5F).

Since TNF signaling predominantly activates NF‐κB to promote tumor cell survival and proliferation [26, 27], we analyzed scRNA‐seq and DSP data, revealing significant NF‐κB pathway enrichment in mregDCs from relapsed tumors compared to those from primary tumors (Figure 5G,H). To further delineate the specific NF‐κB signaling mechanisms involved, we investigated both classical (canonical) and alternative (non‐canonical) NF‐κB pathways [28]. Western blot analysis of key pathway components revealed that TNFR2 blockade significantly suppressed the expression of non‐canonical pathway markers (p52 and RelB) and reduced IKK‐α phosphorylation, while simultaneously decreasing the expression of mregDC markers including PD‐L1 (Figure 5I,J). Using pathway‐specific inhibitors (Sn52 for non‐canonical; Jsh23 for canonical), we demonstrated that p52‐mediated non‐canonical signaling predominantly drives mregDC development (Figure 5K).

Our study revealed that mregDC differentiation was regulated by the TNFR2‐non‐canonical NF‐κB axis, uncovering new opportunities for synergistic anti‐TNFR2/anti‐PD‐L1 combination immunotherapy.

### 2.6 mregDC Recruited CD161^+^CD8^+^ T Cells Vis CCL20‐CCR6 Axis

**FIGURE 6:**
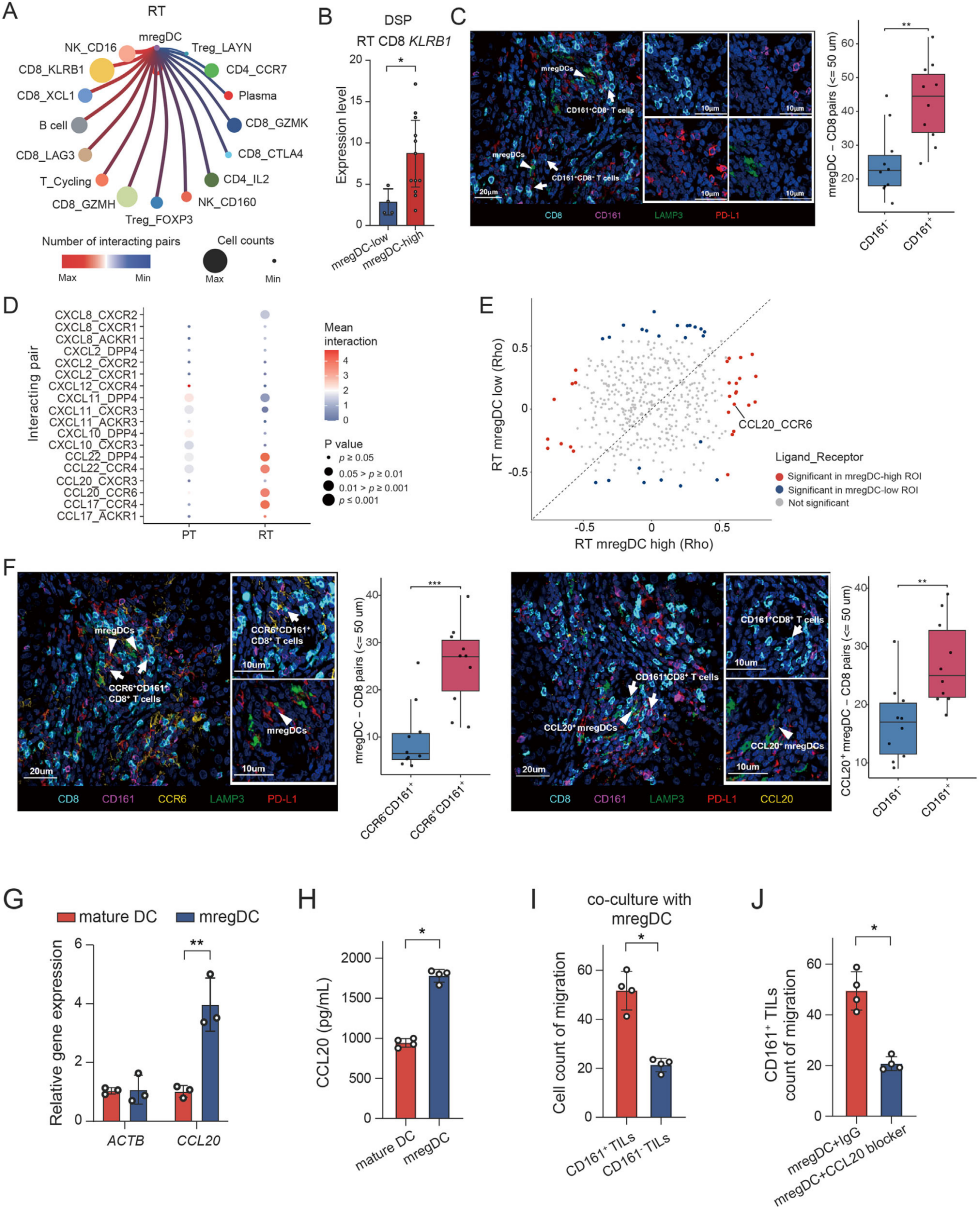
CD161^+^CD8^+^ T cells were recruited by mregDCs through CCL20‐CCR6 axis. (A) Potential intercellular communications based on ligand‐receptor pairs between mregDC and other lymphoid cells in RT samples. The color of lines indicated the number of interacting pairs. The size of dots represents the relative cell counts. (B) Boxplot showing the comparison of *KLRB1* expression level in CD8^+^ T cells between mregDC‐high and mregDC‐low samples by DSP (*n* = 4 in mregDC‐low group, *n* = 12 in mregDC‐high group). (C) Representative multiplex immunofluorescence images of CD161^+^CD8^+^ T cells and LAMP3^+^PD‐L1^+^ mregDCs. Scale bar, 20 µm (left panel). Counts of mregDC within a 50 µm radius of CD161^+^ or CD161^−^ CD8^+^ cells in the FFPE samples (right panel, *n* = 10 per group). (D‐E) Overview of the chemokine‐receptor pairs between PTs and RTs by scRNA‐seq (D) and DSP (E). For scRNA‐seq analysis, color represents the average expression level of interactions between the chemokine and its receptor, and dot size denotes the statistical significance of interaction pairs. (F) Representative multiplex immunofluorescence images of the existence, and close proximity of CCR6^+^CD161^+^CD8^+^ T cells and CCL20^+^ mregDCs. Scale bar, 20 µm. Counts of mregDC within a 50 µm radius of CCR6^+^ or CCR6^−^ CD161^+^ CD8^+^ T cells, and counts of CD161^+^ CD8^+^ T cells within a 50 µm radius of CCL20^+^ or CCL20^−^ mregDCs in the FFPE samples (*n* = 10 per group). (G) Bar plot showing the comparison of relative expression of CCL20 between mature DC and mregDC by qRT‐PCR (*n* = 3 per group). (H) Bar plot showing the comparison of CCL20 concentration between mature DC and mregDC by ELISA (*n* = 4 per group). (I) Bar plot showing the cell count of migrating CD161^+^ or CD161^−^CD8^+^ T cells co‐cultured with mregDC (*n* = 4 per group). (J) Bar plot showing the cell count of CD161^+^CD8^+^ T cells recruited by mregDCs, with CCL20 blocker treatment or not (*n* = 4 per group).

To comprehensively characterize the cellular crosstalk between mregDCs and tumor‐infiltrating lymphocytes, we employed CellChat analysis to map potential ligand‐receptor interactions. Quantitative assessment revealed that mregDCs displayed the strongest interaction signatures with three distinct lymphocyte subsets: NK_CD16 cells, CD8_KLRB1 (CD161) T cells, and CD8_XCL1 T cells (Figure 6A). Digital spatial profiling of CD8^+^ T cell‐enriched regions in recurrent tumors demonstrated significantly elevated *KLRB1* expression in mregDC‐high compared to mregDC‐low AOIs (Figure 6B), consistent with our prior identification of this unique T cell population [15]. Gene signature analysis confirmed marked enrichment of CD161^+^CD8^+^ T cell markers in CD8^+^ AOIs from mregDC‐high AOIs (Figure S6A). Multiplex immunofluorescence analysis of early‐relapse HCC specimens revealed spatial co‐localization of mregDCs with CD161^+^CD8^+^ T cells (Figure 6C), implying potential functional interactions. Quantitative spatial pairing analysis further substantiated this observation, demonstrating a significantly enriched frequency of mregDC–CD161^+^CD8^+^ T cell juxtaposition compared to mregDC–CD161^−^CD8^+^ T cell interactions (Figure 6C). Systematic analysis of chemokine signaling pathways from our single‐cell RNA sequencing data identified the CCL20‐CCR6 axis as the most significantly upregulated chemokine‐receptor pair in recurrent versus primary tumors (Figure 6D). This finding was independently validated through DSP‐based differential interaction analysis (Figure 6E), with multiplex IF staining demonstrating close proximity between CCL20^+^ mregDCs and CCR6^+^CD161^+^CD8^+^ T cells (Figure 6F), with quantitative image analysis showing significantly higher frequencies of CCL20^+^ mregDC–CD161^+^CD8^+^ and mregDC–CCR6^+^CD161^+^CD8^+^ pairs within 50 µm in recurrent tumors compared to their respective controls (Figure 6F).

To elucidate the mechanisms underlying CD161^+^CD8^+^ T cell recruitment by mregDCs, we examined CCL20 expression at both transcriptional and protein levels. PCR analysis showed significantly elevated CCL20 mRNA expression in mregDCs compared to mature DCs (Figure 6G). Consistent with this finding, ELISA analysis demonstrated enhanced CCL20 secretion by mregDCs relative to mature DCs (Figure 6H). To functionally validate the chemotactic effect of mregDCs, we performed co‐culture experiments which demonstrated preferential recruitment of CD161^+^CD8^+^ T cells over CD161^−^ populations by mregDC (Figure 6I). Neutralizing antibody experiments confirmed the specificity of this interaction, as CCL20 blockade markedly reduced mregDC‐mediated CD161^+^CD8^+^ T cell migration (Figure 6J).

Together, these findings mechanistically delineate mregDC‐CD161^+^CD8^+^ T cell crosstalk via the CCL20‐CCR6 axis.

### 2.7 MDSC‐Like FCN1^+^ Monocytes Might Be Recruited by mregDC to Drive Immunosuppression in Relapsed HCC

**FIGURE 7:**
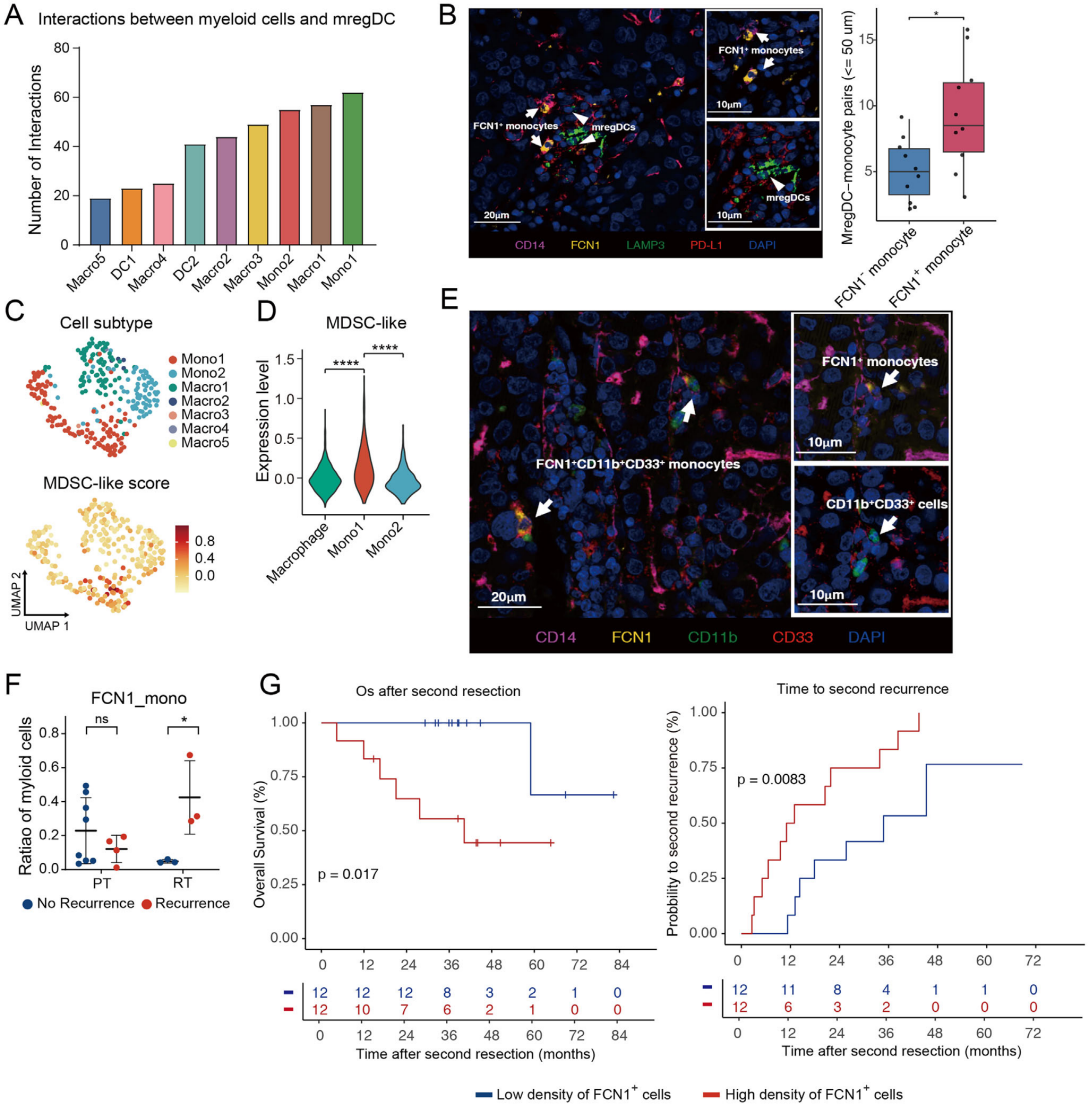
FCN1^+^ monocyte is most closely associated with mregDC in myeloid cell populations of early‐relapse HCC. (A) Bar plot showing the number of interactions predicted by CellChat. (B) Representative multiplex immunofluorescence images of neighboring locations of FCN1^+^ monocytes and mregDCs. Scale bar, 20µm (left panel). Counts of mregDC within a 50 µm radius of FCN1^+^ or FCN1^−^ monocytes in the FFPE samples (right panel, *n* = 10 per group). (C) UMAP plots of monocytes and macrophages cell distribution (upper panel), and the MDSC‐like signature score for each cell (bottom panel). (D) Violin plot showing expression level of MDSC‐like score among macrophages and two monocyte subsets. (E) Representative multicolor fluorescent images of the existence of MDSC‐like FCN1^+^ monocytes (FCN1^+^CD11b^+^CD33^−^). Scale bar, 20 µm. (F) Dot plots showing the fraction of FCN1^+^ monocytes in recurrent or non‐recurrent group after resection of PT or RT, respectively (8 patients in no recurrence group, 4 patients in recurrence group). (G) Kaplan‐Meier analyses showing the overall survival (left panel), and time‐to‐recurrence (right panel) of patients with recurrent HCC, characterized by high or low density of FCN1^+^ cells. Survival analyses were compared using the log‐rank tests (*n* = 24).

To further investigate the associations between mregDCs and other myeloid cells, we quantified potential ligand‐receptor pairs using CellChat analysis. Results showed that mregDCs had the greatest number of ligand‐receptor pairs with the Mono1 cluster (FCN1^+^ monocytes) (Figure 7A). Multiplexed immunofluorescence assays confirmed close proximity between FCN1^+^ monocytes and mregDCs (Figure 7B). Quantitative analysis further demonstrated that mregDC–FCN1^+^ monocyte pairs within 50 µm were significantly more frequent than mregDC–FCN1^−^ monocyte pairs (*p* < 0.05), supporting the notion of preferential spatial crosstalk between these two cell populations, supporting their functional crosstalk. We further performed ligand–receptor interaction analysis between mregDCs and FCN1^+^ monocytes based on our single‐cell RNA‐sequencing data. CellChat analysis revealed that mregDCs may communicate with FCN1^+^ monocytes via the LGALS9–PTPRC signaling axis (Figure S7A). We next evaluated the immunosuppressive potential of these monocytes by applying an established MDSC‐like signature [29]. Signature scoring demonstrated predominant expression in FCN1^+^ Mono1 cluster (Figure 7C,D). Multi‐color immunofluorescence validated the MDSC‐like phenotype of FCN1^+^ monocytes (Figure 7E). The relationship between the proportion of FCN1^+^ monocytes and HCC recurrence showed variation among patients with PT and RT. Those RT patients with higher fraction of FCN1^+^ monocytes were more likely to develop a second recurrence (Figure 7F), while FCN1^+^ monocytes showed no prognostic value in PT patients. Kapla‐Meier curve analysis indicated that the enrichment of FCN1^+^ monocytes was significantly associated with a worse overall survival and higher probability of the second recurrence (Figure 7G). In multivariate analysis of our validation cohort, the density of FCN1^+^ monocyte by IHC staining was the independent prognostic factor for overall survival (HR, 5.76; 95% CI, 1.60‐20.72; p = 0.007) and time to recurrence (HR, 2.33; 95% CI, 1.11–4.89; *p* = 0.026) in HCC (Figure S7B,C).

## 3 Discussion

Through comprehensive multi‐omics analysis of the tumor immune microenvironment in early‐relapse HCC, we have identified and characterized a distinct dendritic cell population with immunoregulatory properties that plays a pivotal role in tumor immune evasion. Our integrated single‐cell and spatial transcriptomic approaches have yielded several key insights with important clinical and biological implications.

First, we observed significant enrichment of mregDCs in early‐relapse compared to primary HCC tissues, suggesting their potential involvement in tumor recurrence. Transcriptional profiling revealed these cells exhibit enhanced migratory capacity and maturation status while displaying immunosuppressive signatures. Zhang et al. integrated pan‐cancer single‐cell datasets of dendritic cells and revealed that tumor‐infiltrating *LAMP3* ^+^ DCs are predominantly derived from both cDC1 and cDC2 subsets. Notably, *LAMP3* ^+^ DCs originating from cDC2 exhibited substantially attenuated anti‐tumor capacity compared with those derived from cDC1 [30], which is highly consistent with the conclusions of our study. Importantly, the presence of mregDCs strongly correlated with poor clinical outcomes, aligning with previous reports associating these specific DC phenotypes with unfavorable prognosis across multiple cancer types [31, 32].

Beyond correlative observations, we explored the upstream regulatory factors underlying the phenotypic emergence of mregDC. Metabolic programs driven by tumor cell‐derived lactate are established as key mediators inducing the mregDC phenotype. Hanks et al. further demonstrated that fatty acid oxidation contributes to the maturation and maintenance of the immunosuppressive state of mregDCs [33]. Nevertheless, the functional roles of immune‐related components within the tumor microenvironment, particularly cytokines, in driving the emergence of mregDCs remain poorly defined. In this study, we revealed the central role of TNF signaling in regulating mregDC development. While macrophages are well recognized as one of the primary sources of TNF‑α, previous studies have demonstrated that tumor‑infiltrating macrophages in the early recurrent HCC microenvironment are predominantly of the M2‑like phenotype [15]. Notably, M2 macrophages exhibit markedly diminished TNF‑α secretion capacity relative to their M1 counterparts [34]. We demonstrated that TNF‐α derived from tumor‐infiltrating CD161^+^CD8^+^ T cells drives DC differentiation toward an mregDC phenotype characterized by upregulated immunoregulatory molecules. This observation extends previous reports of TNF's role in DC maturation by identifying TNFR2 as a critical mediator [35]. Our finding that TNFR2 blockade can reverse the mregDC phenotype suggests therapeutic potential for targeting this pathway in HCC. While immune checkpoint blockade has revolutionized cancer treatment, persistent challenges remain in treating non‐responsive tumors, likely due to profound microenvironmental immunosuppression [36]. In this context, TNFR2, one of two receptors mediating TNF biological activity [37], has emerged as an important regulator of tumor immunosuppression [38, 39, 40], with recent studies highlighting its potential as a therapeutic target through either agonistic antibody to enhance effector T cell function or antagonists to disrupt regulatory T cell activity [41, 42].

The clinical utility of PD‐L1 as a biomarker remains limited by technical challenges including heterogeneous expression patterns and assay variability [43, 44, 45, 46]. Our discovery that PD‐L1 expression is dynamically regulated during mregDC development through non‐canonical NF‐κB pathway activation provides a novel mechanistic insight. This finding raises important questions about the relative contributions of intratumoral versus lymphoid organ DC populations in mediating responses to PD‐1/PD‐L1 blockade [47, 48, 49, 50, 51, 52, 53], particularly given reports that LAMP3^+^ DCs may predict better response to immune checkpoint therapy [13, 54], though the role of PD‐L1 expression on these cells requires further investigation. Non‐canonical NF‐κB signaling is primarily activated by TNF family ligands and plays a key role in regulating the maturation and immunosuppressive phenotype of mregDCs. For the clinical implementation of inhibiting non‐canonical NF‐κB signaling in mregDCs, we will further focus on cell‐specific targeting strategies to avoid off‐target effects on normal cells. One feasible approach is to develop mregDC‐specific delivery systems, such as lipid nanoparticles loaded with small interfering RNAs targeting key molecules of the non‐canonical NF‐κB pathway [55].

Additionally, our study provides mechanistic evidence that mregDCs actively contribute to establishing an immunosuppressive tumor microenvironment. Mao et al. demonstrated in a murine colon cancer model that mregDCs establish intimate crosstalk with Tregs via the CCL17–CCR4 chemotactic axis, thereby facilitating immune escape of tumor cells [17]. Our work also elucidates the complex cellular interactions within the microenvironment of early relapse hepatocellular carcinoma. We demonstrate that mregDCs recruit innate‐like CD161^+^CD8^+^ T cells through CCL20 secretion, forming dysfunctional immune cell clusters that facilitate immune escape, a finding consistent with established roles of DCs in promoting immune tolerance [56, 57]. This finding offers a plausible biological explanation for the association between mregDC accumulation and accelerated disease progression while identifying potential therapeutic targets within this pathway. Regarding the clinical implementation of blocking the CCL20‐CCR6 axis, we propose two feasible strategies based on the experimental results of this study and existing clinical research evidence. On one hand, neutralizing antibodies targeting CCL20 or CCR6 can be developed and applied in clinical practice. On the other hand, small‐molecule inhibitors targeting CCR6 can be developed, which have the advantages of oral administration, good tissue penetration, and convenient clinical application compared with neutralizing antibodies. Furthermore, we identified potential cooperation between mregDCs and MDSC‐like FCN1^+^ monocytes, cell types previously associated with immune‐desert tumors [58], suggesting coordinated mechanisms of immune suppression.

Several limitations warrant consideration. Our single‐cell analysis, while informative, may not fully capture HCC heterogeneity due to sample size constraints. The cross‐sectional design precludes direct observation of temporal immune cell dynamics. While mouse model results are promising, there was no mouse model that fully simulates the early recurrence of hepatocellular carcinoma in this study, and clinical validation in larger cohorts is needed.

In conclusion, this study provides fundamental insights into mregDC biology in HCC recurrence and identifies the TNF‐TNFR2 axis as a potential therapeutic target. Future studies should focus on elucidating detailed mechanisms of mregDC‐mediated immunosuppression, exploring combination strategies with existing immunotherapies, and validating these findings in clinical settings to advance precision immunotherapy for HCC.

## 4 Experimental Section

### 4.1 Study Cohorts

The discovery cohort was constituted by collecting paired fresh tumor samples and adjacent normal tissue samples (at least 2 cm away from the matched tumor tissues) from 6 HCC patients with early relapse, defined as recurrence within two years after curative resection, and 12 treatment‐naive primary HCC patients. For the validation of the MIHC cohort, paired available FFPE tumor samples and adjacent normal tissue samples were collected from 41 patients with early relapsed and primary HCC. Among these, 24 patients with available prognostic data after surgical resection for recurrent HCC were included in the prognostic analysis. Additionally, two representative cases with sufficient ROIs in both primary tumor and early‐relapse tumor tissues were selected from paired PT/RT samples, with 42 AOIs manually annotated for spatial analysis. Detailed clinical and pathological information for these patients is presented in our previous publication [15]. Informed consent was obtained from all patients for the collection of clinical information, tissue collection, and research testing, in accordance with Institutional Review Board (IRB)‐approved protocols (B2019‐060R) at Zhongshan Hospital, Fudan University.

### 4.2 Immunohistochemical Staining and Digital Pathology Analysis

FFPE tissue samples used for immunohistochemical staining were from our validation cohort. All the staining process was carried out on an IHC/ISH System (BenchMark Ultra, Roche) following the manufacturer's instruction. The whole sections were analyzed using a digital pathology system (HALO 3.3, Indica Labs). The average density (cells/mm^2^) of positive cells were quantitatively scored. The number of target cells within a 50 µm radius of various cell types was automatically quantified by computer.

### 4.3 Multiplex Immunofluorescence Analysis

Multiplexed immunofluorescence staining was performed using 3‐µm‐thick sections of FFPE tissues in accordance with standard protocols at Nanjing Freethinking Biotechnology Co., Ltd. (China). Briefly, after being deparaffinized and dehydrated, slides were immersed in Tris‐EDTA antigen retrieval buffer, and heat‐induced antigen retrieval was performed. To block endogenous peroxidases and antigens, 3% H2O2 and 3% bovine serum albumin were used, respectively. The slides were then sequentially incubated with primary antibodies. Primary antibodies were sequentially applied, followed by horseradish peroxidase‐conjugated secondary antibody incubation and tyramide signal amplification, and finally the nuclei were counterstained with DAPI. A Pannoramic MIDI imaging system (3D HISTECH) was used to scan the stained slides. The images were analyzed and quantified by HALO.

### 4.4 Single‐Cell Isolation

Fresh tumor and adjacent normal tissue samples were surgically removed and immediately placed in transport medium (90% DMEM, 10% FBS; GIBCO) and transferred to the laboratory in a refrigerated container. Suitable tissue blocks (free from necrotic/hemorrhagic foci and minimal fibrous tissue) were cut into 1–3 mm pieces and transferred to gentle MACS C Tubes (Miltenyi Biotec) containing 5 mL of digestive enzyme from the Tumor Dissociation Kit (Miltenyi Biotec). Tissue dissociation was performed using the gentleMACS Dissociator (Miltenyi Biotec) following this protocol: initial mechanical disruption, 30‐min incubation at 37°C on a shaker, second mechanical disruption, another 30‐min incubation, final mechanical disruption. The resulting suspension was filtered through a 70 µm filter with 2% FBS added. The single‐cell suspension was centrifuged at 400 g for 7 min and resuspended in CELLSAVING medium (NCM biotech) for further experiments.

### 4.5 Flow Cytometry and Cell Sorting

Single‐cell suspensions were prepared from murine or human tumor specimens. For surface staining, cells were incubated with Fc Block (BD Biosciences) for 10 min at 4°C to prevent non‐specific binding, then stained with fluorophore‐conjugated antibodies in PBS containing 2% FBS for 30 min at 4°C. Dead cells were excluded using Zombie Aqua Fixable Viability Dye (BioLegend). Flow cytometry data were acquired on a BD LSRFortessa analyzer, and cell sorting was performed on a BD FACSAria Fusion SORP cell sorter. The target cells were sorted into 15 mL conical tubes pre‐coated with FBS and containing complete medium.

### 4.6 Western Blot

Cell pellets were collected and lysed by RIPA buffer (Beyotime) with protease inhibitor cocktail (APExBIO) and phosphatase inhibitor cocktails (Yeasen). Protein quantity was quantified by Pierce BCA Protein Assay Kit (Thermo Fisher), then equal amounts of protein (5‐10 µg for histones, 10–20 µg for other proteins) were run on 12.5% or 10% SDS‐PAGE gels (Epizyme). Membranes were blocked in 5% nonfat dry milk dissolved by TBST for 1 h at room temperature and incubated with primary antibodies at 4°C overnight. After incubation with HRP‐conjugated secondary antibodies for 1 h at room temperature, membranes were visualized by the ChemiDoc Imaging System (Bio‐rad).

### 4.7 RNA Isolation and Reverse Transcription‐Quantitative PCR (RT‐qPCR)

Cellular RNA was extracted using TRIzol reagent (Invitrogen; Thermo Fisher Scientific, Inc.). Complementary DNA synthesis was carried out using the PrimeScriptTM reverse transcriptase reagent kit (Takara Bio, Inc.). Amplification and quantification were conducted using the ABI PRISM 7900 Sequence Detection System (Applied Biosystems; Thermo Fisher Scientific, Inc.) and SYBR Premix Ex TaqTM (Tli RNaseH Plus; Takara Bio, Inc.). The expression level of the target gene was normalized to that of β‐actin and the 2‐ΔΔCq method.

### 4.8 ELISA

The supernatants were analyzed using ELISA. ELISA kits for CCL20 and TNF‐α were sourced from MULTISCIENCES, and ELISA assays were performed following the manufacturer's instructions.

### 4.9 Ex Vivo Cell Induction Assay

Human CD14^+^ monocytes were isolated from peripheral blood mononuclear cells (PBMCs) of healthy donors with the CD14 MicroBeads (Miltenyi Biotec) and Ficoll‐Paque Plus (Lympholyte Cedarlane) centrifugation following the manufacturer's instructions. Purified CD14^+^ monocytes were then cultured in the presence of human GM‐CSF and human IL‐4 (Peprotech) for 7 days. Differentiated cells were plated at 200,000 cells per well in 24 flat bottom tissue culture treated plates (Corning) and stimulated for 24 h with TNF‐α (5 ng mL^−1^) to induce mregDCs.

Murine bone marrow was isolated from C57BL/6 mice, and monocytes were subsequently enriched by negative selection using a monocyte isolation kit (Miltenyi) according to the manufacturer's instructions. The isolated monocytes can be cultured for up to 5 d in macrophage medium supplemented with 5 ng mL^−1^ recombinant murine granulocyte‐macrophage colony‐stimulating factor (rmGM‐CSF) and 2.5 ng mL^−1^ recombinant murine interleukin‐4 (rmIL‐4). Monocytes should be seeded at a density of 1×10^6^ cells per well in six‐well plates with a final volume of 2 mL. Then differentiated cells were plated at 200 000 cells per well in 24 flat bottom tissue culture treated plates and stimulated for 24 h with TNF‐α (5 ng mL^−1^) to induce mregDCs.

### 4.10 Ex Vivo Migration Assay

We conducted migration assays by sorted CD8^+^ T cells and conditioned medium from mregDCs or mature DCs. In the transwell migration assay, 96‐well 5‐µm inserts (Corning) were utilized. The lower chamber was loaded with 200 µL of conditioned medium as chemo‐attractants, and CD8^+^ T (5 × 10^4^) suspended in 50 µL^−1^ medium were then placed in the upper chamber. To investigate the biological function of CCL20, 2 µg mL^−1^ neutralizing anti‑CCL20 monoclonal antibody (Invitrogen) or isotype control antibody was added to the ex vivo cell recruitment assay system. After 3 h of incubation at 37°C under 5% CO_2_ conditions, migrated and unmigrated CD8^+^ T cells were collected from the lower and upper chambers, respectively. Cell number quantification was accomplished using counting beads for FACS (Invitrogen), and the migration ratio was determined by dividing the number of migrated cells by the sum of migrated and immigrated cells.

### 4.11 In Vivo Assays

Subcutaneous tumor models were established by subcutaneous inoculation of Hepa1‑6 cells into NOD/SCID mice (2 × 10^6^ cells per mouse). After subcutaneous tumor formation, necrotic regions were removed, and tumor fragments measuring 2–3 mm^3^ were orthotopically implanted into the left liver lobe of C57BL/6 mice to generate orthotopic HCC models. One week following orthotopic implantation, C57BL/6 mice were randomly divided into five treatment groups. Group 1 received intratumoral injection of 2 × 10^5^ murine mature DCs resuspended in 50 µL PBS, administered once weekly for three consecutive weeks. Group 2 received intratumoral injection of 2 × 10^5^ murine mregDCs in 50 µL PBS, once weekly for three weeks. Group 3 received intratumoral mregDC administration combined with intraperitoneal injection of anti‑TNFR2 antibody (100 µg per mouse, three times weekly for three weeks, Invitrogen). Group 4 received intratumoral mregDCs together with intraperitoneal anti‑PD‑L1 antibody (200 µg per mouse, three times weekly for three weeks, BioXCell). Group 5 was treated with intratumoral mregDCs plus combined intraperitoneal administration of anti‑PD‑L1 and anti‑TNFR2 antibodies. All mice were euthanized and liver tissues collected at four weeks after orthotopic tumor engraftment as the experimental endpoint.

We subcutaneously inoculated C57BL/6 mice with a mixture of Hepa1‐6 cells (5 × 10^5^ cells per mouse) and pre‐prepared murine mature DC or mregDCs. Subsequently, murine mature DCs or mregDCs were administered via intratumoral injection (2 × 10^5^ cells in 50 µL PBS per mouse), once per week. Tumor volume was measured regularly post‐inoculation. Different drug treatment groups were established, wherein mice received intraperitoneal injections of PD‐L1 neutralizing antibody, TNFR2 neutralizing antibody, or isotype control antibody following tumor cell inoculation. In addition, to investigate the role of TNF‐α in inducing the mregDC phenotype, subcutaneous tumors received intratumoral injection of recombinant murine TNF‐α protein (5 µg in 50 µL PBS per mouse, Selleck) or PBS every other day, serving as the treated and control groups, respectively. Tumor volumes across all groups were monitored periodically.

All animal protocols in this study were reviewed and approved by the Animal Care and Use Committee of the Department of Laboratory Animal Science, Fudan University (No. 202309025S).

### 4.12 Differentially Expressed Gene Analysis for PT and RT mregDs

To identify characteristic genes in mregDs cells from RT, we conducted a differential expression analysis comparing mregDs cells isolated from PT and RT. This analysis was performed using the “FindMarkers” function available in the Seurat package. Genes were considered differentially expressed when they exhibited a log‐scaled fold change of 0.25 or greater and a *p* value less than 0.05, as determined by the Wilcoxon Rank Sum test.

### 4.13 Single‐Cell RNA‐Seq Analysis

Raw sequencing data were processed with the Cell Ranger pipeline (10x Genomics) to generate gene–cell count matrices. Subsequent analyses were performed in Scanpy. Low‐quality cells with low transcript complexity, abnormally high gene counts (potential multiplets), or excessive mitochondrial RNA content were excluded. After normalization, log‐transformation, and identification of highly variable genes, expression values were scaled and subjected to principal component analysis (PCA). Putative doublets were identified using Scrublet and removed prior to dimensionality reduction and clustering. Uniform manifold approximation and projection (UMAP) was used for visualization, and clusters were defined using the Leiden algorithm. Cell type annotation was based on canonical marker gene expression.

### 4.14 Single‐Cell ssGSEA for Different Subpopulations

We performed single‐sample Gene Set Enrichment Analysis (ssGSEA) to evaluate gene set enrichment in individual cells across different subpopulations or samples. The analysis was conducted using the GSVA package in R. Gene expression data for each cell was first normalized to account for sequencing depth differences. Pre‐defined gene sets were selected from You's work [17]. The ssGSEA algorithm was applied to each cell's expression profile using the “gsva” function with the “ssgsea” method. This process calculated enrichment scores for each gene set in every cell. The resulting scores were then aggregated by cell subpopulations for comparison. Statistical tests, including Wilcoxon rank‐sum test, were applied to assess significant differences in enrichment scores between subpopulations. The results were visualized using violin plots to illustrate pathway activity differences across cell subsets.

### 4.15 Cell‐Cell Communication Analysis

The potential intercellular communication network of cell populations was conducted by CellChat. The expression matrix normalized through Seurat was imported, and a CellChat object was generated utilizing the create CellChat function. Subsequently, the identify over expressed genes and identify over expressed interaction functions were employed to detect over‐expressed ligands or receptors.

### 4.16 TCGA Data Analysis

The results of prognosis were based on the data generated by TCGA Research Network (https://www.cancer.gov/tcga). Samples with available data and certain histological types were enrolled. Survival analysis was conducted by Kaplan‐Meier with p value calculated by a log rank test using R package survminer (ref) and survival (ref). Cases with absent or value zero data of overall survival or disease‐free interval were removed.

### 4.17 GeoMx DSP

Spatial transcriptomics analysis was performed using the GeoMx Digital Spatial Profiler (DSP) platform (NanoString Technologies) according to the manufacturer's instructions. Briefly, 5 µm sections of formalin‐fixed paraffin‐embedded (FFPE) tissue were subjected to deparaffinization and antigen retrieval. The sections were then incubated with a cocktail of fluorescently labeled antibodies serving as morphology markers (pan‐cytokeratin for tumor cells, CD45 for immune cells, CD11c for dendritic cells, and SYTO13 for nucleic acids) and photocleavable oligonucleotide‐labeled primary antibodies (profiling antibodies). In situ hybridization was conducted using the GeoMx Whole Transcriptome Atlas Panel (WTA), encompassing 18,677 genes.

After staining, the slides were loaded onto the GeoMx DSP instrument for high‐resolution fluorescence imaging. Circular regions of interest (ROIs) with a maximum diameter of 660 µm were defined on each slide, focusing on intratumoral mregDC‐enriched areas distributed across the tissue. Within each ROI, molecularly defined tissue compartments were established through fluorescent marker colocalization to enable compartment‐specific transcriptional profiling. In total, 42 ROIs were identified across 4 FFPE specimens. Once ROIs were selected, the GeoMx DSP system employed UV‐mediated photocleavage to release the barcoded RNA probes from their respective tissue regions. The liberated probes from individual segmented areas were then collected into separate wells of a 96‐well plate. Samples were then transferred for probe hybridization and PCR amplification. Purified and quality‐checked products were sequenced on an Illumina NovaSeq 6000 platform.

NovaSeq6000‐generated GeoMx WTA sequencing reads were compiled into ROI‐specific FASTQ files, which were then converted to digital count files using the NanoString GeoMx NGS DnD Pipeline.

### 4.18 Statistical Analysis

Statistical analyses were performed using SPSS version 23 for windows (IBM, Armonk, NY, USA). Data are presented as means and standard deviations or medians and ranges. The Student's t test or Mann‐Whitney U test was used to examine differences between two groups. Kaplan‐Meier survival curves and a log‐rank testing were performed to estimate the prognostic significance. Two‐sided *P*<0.05 was considered statistically significant.

## Author Contributions

Z.Z., Y.Z., L.W., and Y.S. conceived and designed the project, Z.Z., L.D., B.W., and Y.S. collected the clinical and animal samples, Z.Z., L.D., H.F., and L.H. performed the experiments, L.D, Y.Z., L.H., W.H., and C.W. curated bioinformatics analysis, Y.Z., W.H., C.W., A.Ö., and L.W. processed data, Z.Z. and L.D. performed histopathological analysis of tissues, Z.Z., L.D., Y.Z., and Y.S. interpreted the data, Z.Z., L.D., Y.Z., H.F., L.H., L.W., and Y.S. wrote the draft. J.Z., J.F., W.G., L.W., and Y.S. supervised the project.

## Conflicts of Interest

The authors declare no conflicts of interest.

## Supporting information




**Supporting File**: advs75695‐sup‐0001‐SuppMat.pdf.

## Data Availability

The data that support the findings of this study are available on request from the corresponding author. The data are not publicly available due to privacy or ethical restrictions.
