# Supplementary material for: Tumor‐Infiltrating mregDCs Restrain Anti‐Tumor Immunity in Early Relapse HCC
Source: Adv Sci (Weinh). 2026 May 14:e75695. Online ahead of print. doi: 10.1002/advs.75695 (PMC13335829; doi:10.1002/advs.75695)
Supplement: Supplementary file 1 — Supporting File: advs75695‐sup‐0001‐SuppMat.pdf. [file ADVS-9999-e75695-s001.pdf]

Tumor-infiltrating mregDCs restrain anti-tumor immunity in early relapse HCC

Zefan Zhang[1, 2, 10], Lin Ding[3, 10], Yu Zhong[4, 5, 10], Haokang Feng[1, 2, 10], Linglong Huang[6, 10], Waidong Huang[4, 7], Chunqing Wang[4, 7, 8], Arne Östman[6], Beili Wang[3], Jian Zhou[1], Jia Fan[1], Wei Guo[3, 11], Liang Wu[2, 4, 9,11], Yunfan Sun[1, 2, 11]

Affiliations:

[1] Department of Hepatobiliary Surgery and Liver Transplantation, Liver Cancer Institute, Zhongshan Hospital, Fudan University; Key Laboratory of Carcinogenesis and Cancer Invasion, Ministry of Education, Shanghai 200032, China.

[2] Zhongshan-BGI Precision Medical Center, Zhongshan Hospital, Fudan University, Shanghai 200032, China.

[3] Department of Laboratory Medicine, Zhongshan Hospital, Fudan University, Shanghai 200032, China.

[4] BGI Research, Chongqing 401329, China.

[5] Department of Pathology, College of Basic Medicine, Chongqing Medical University, Chongqing 400016, China

[6] Department of Oncology-Pathology, Karolinska Institutet, Stockholm, Sweden

[7] College of Life Sciences, University of Chinese Academy of Sciences, Beijing 100049, China

[8] Department of Pathology, College of Basic Medicine, Chongqing Medical University, Chongqing 400016, China.

[9] State Key Laboratory of Genome and Multi-omics Technologies，BGI Research，Shenzhen 518083, China

[10] These authors contributed equally.

[11] Corresponding author: Yunfan Sun, Department of Hepatobiliary Surgery and Liver Transplantation, Liver Cancer Institute, Zhongshan Hospital, Fudan University; Key Laboratory of Carcinogenesis and Cancer Invasion, Ministry of Education, Shanghai 200032, China, yunfan_sun@msn.com. Liang Wu, State Key Laboratory of Genome and Multi-omics Technologies，BGI Research，Shenzhen 518083, China; BGI Research, Chongqing 401329, China, wuliang@genomics.cn. Wei Guo, Department of Laboratory Medicine, Zhongshan Hospital, Fudan University, Shanghai 200032, China, guo.wei@zs-hospital.sh.cn.

Supplementary Figure 1

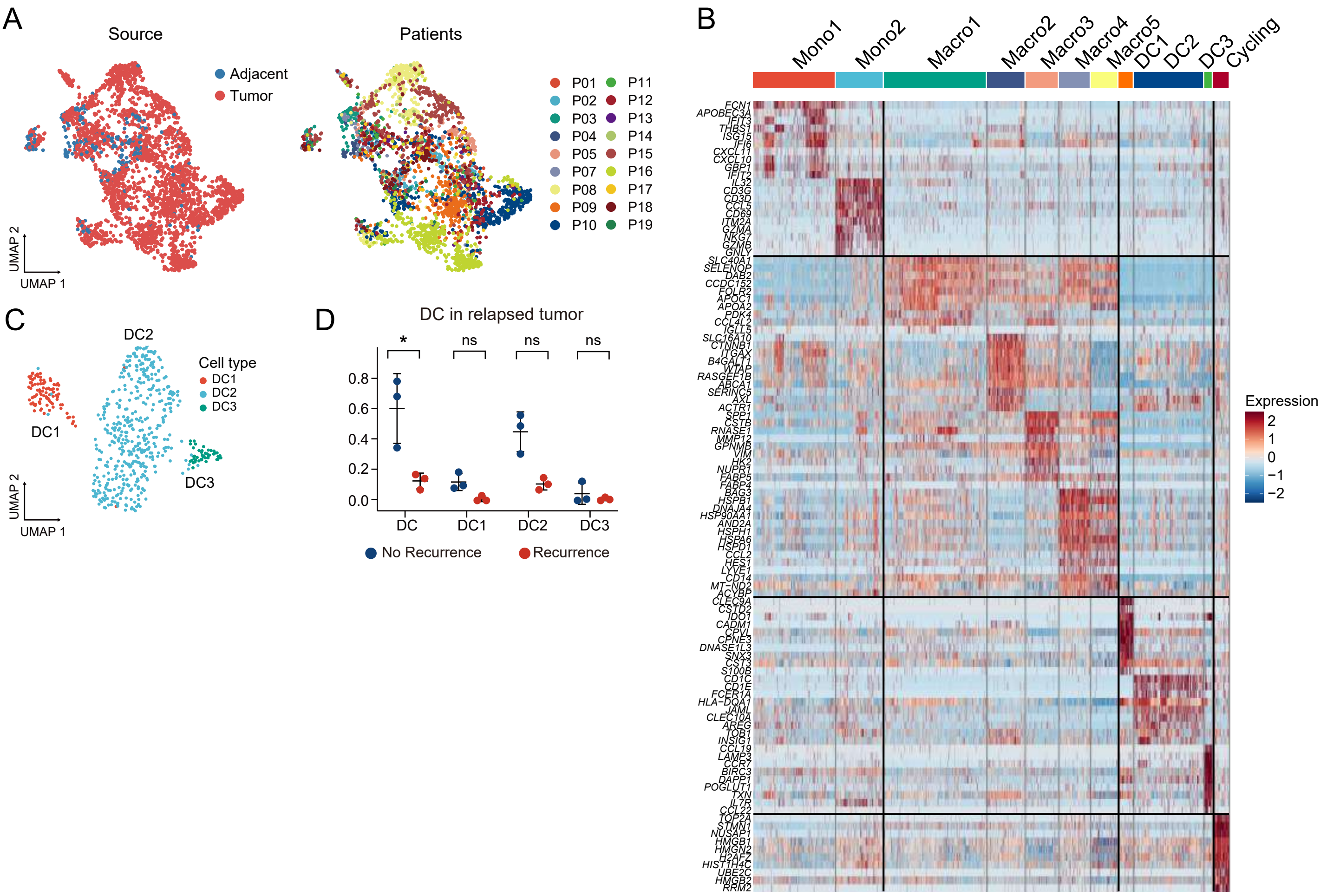

Supplementary figure 1. Single-cell profiling of myeloid cells in primary and early-relapse HCC, related to Figure 1.

(A) The UMAP plot provides a visual representation of myeloid cell aggregates in 18 patients according to cell type (top panel) and patient origin (bottom panel).

(B) Heatmap showing the comparison of specific gene expression patterns within distinct cell types. The top bars provide annotations for each cluster.

(C) UMAP visualization of three DC subsets.

(D) Scatterplots showing the distinct ratio of DCs in recurrent or non-recurrent group after resection of RT (8 patients in no recurrence group, 4 patients in recurrence group).

Supplementary Figure 2

A

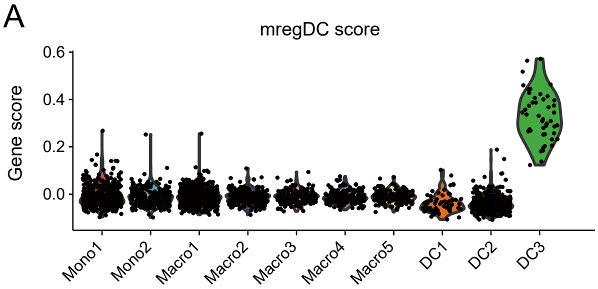

Supplementary figure 2. Differentiation characteristics of DC3s, related to Figure 2.

(A) Violin plot indicating the difference in the mregDC score among myeloid cells.

Supplementary Figure 3

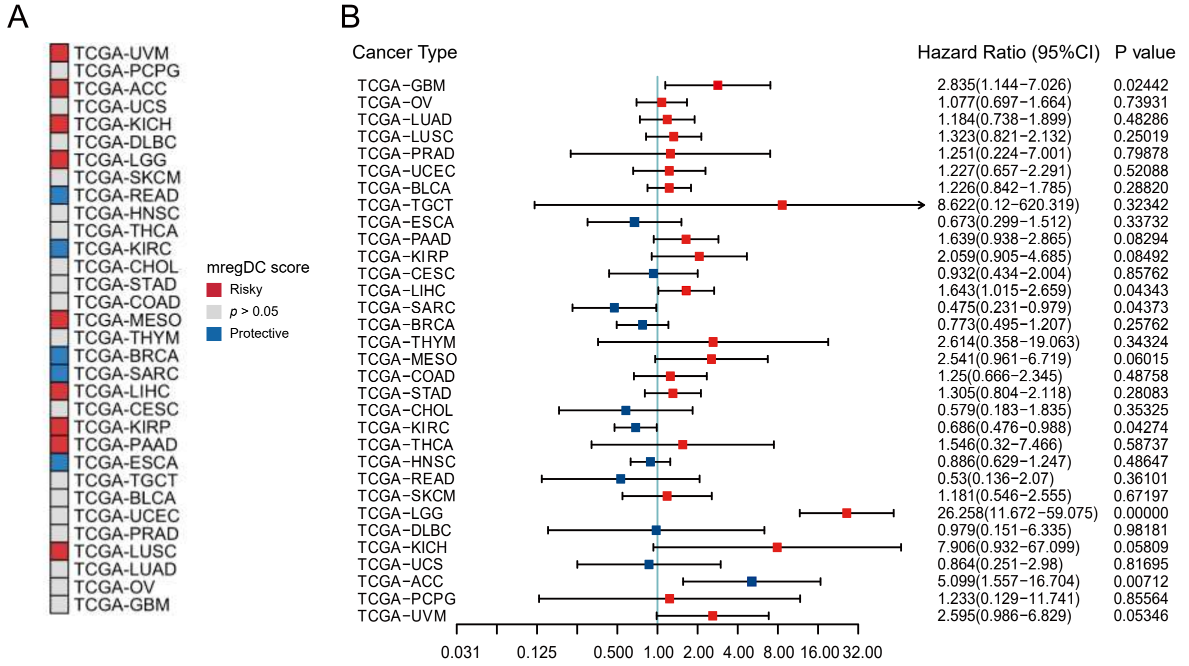

Supplementary figure 3. Pan-cancer analysis of mregDC scores and patient survival in TCGA cohorts, related to Figure 3.

(A) Heatmaps showing the relationship between mregDC scores and the prognosis of differnet cancer types from the TCGA cohort by log-rank tests.

(B) Forest plot of hazard ratios investigating the relationship between mregDC scores and the prognosis of differnet cancer types from the TCGA cohort by Cox proportional hazards model.

Supplementary Figure 4

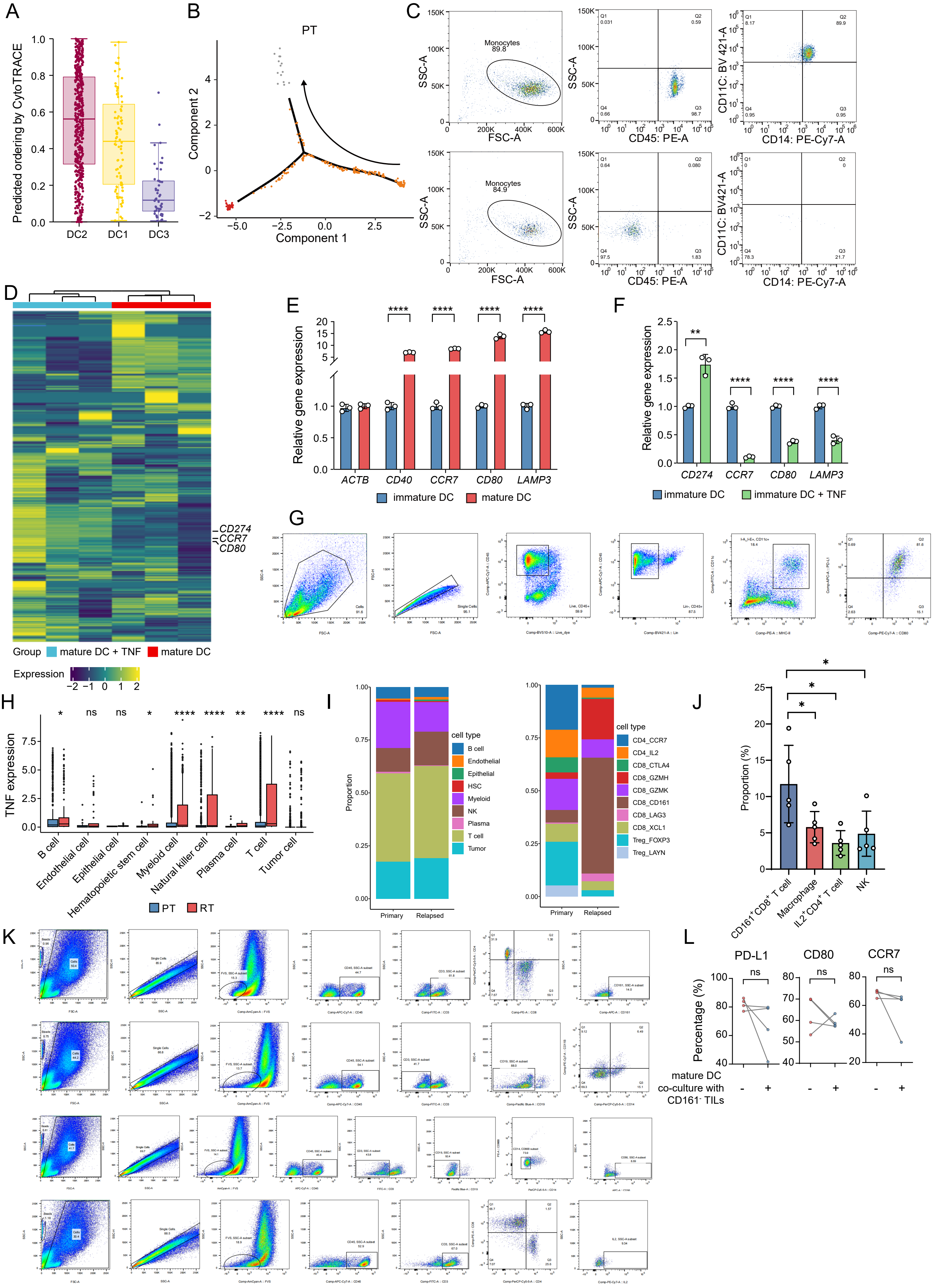

Supplementary figure 4. TNF-α promotes the transition from mature DCs to mregDCs, related to Figure 4.

(A) Box plot illustrating the predicted ordering by CytoTRACE.

(B) Monocle2-based developmental trajectory analysis revealing DC3 potentially differentiated from both DC1 and DC2 in PT.

(C) Flow cytometric characterization of DC maturation markers in the *ex vivo* differentiation model.

(D) Heatmap showing the comparison of specific gene expression patterns between mature DCs and mature DCs treated with TNF-α. Columns denote groups; rows denote genes.

(E) Box plot illustrating the mregDC-specific gene expression in immature DCs and mature DCs. The relative expression levels of the indicated genes were determined by qRT-PCR (n=3 per group).

(F) Box plot illustrating the mregDC-specific gene expression in immature DCs and immature DCs treated with TNF-α. The relative expression levels of the indicated genes were determined by qRT-PCR (n=3 per group).

(G) Schematic diagram of the flow cytometry sorting strategy for mregDCs.

(H) Box plot illustrating the *TNF* expression of diverse cell clusters in patients with RT and PT. Significance was determined by unpaired Wilcoxon test.

(I) The proportions of distinct cell subsets among all cells (left panel), and the percentages of different T cell subsets within total T cells (right panel) based on single-cell RNA-seq data.

(J) Proportion of macrophages, NK cells, $IL2^+$ $CD4^+$ T cells and $CD161^+$ $CD8^+$ T cells among total immune cells in tumor tissues obtained from patients with early recurrent HCC based on flow cytometry analysis (n=5 per group).

(K) Schematic of the flow cytometry gating strategy for macrophages, NK cells, $IL2^+$ $CD4^+$ T cells and $CD161^+$ $CD8^+$ T cells detection.

(L) Dot plots comparing PD-L1, CD80, CCR7 expression between mature DCs and mature DCs co-culture with $CD161^-$ $CD8^+$ T cells (n=4 per group).

Supplementary Figure 5

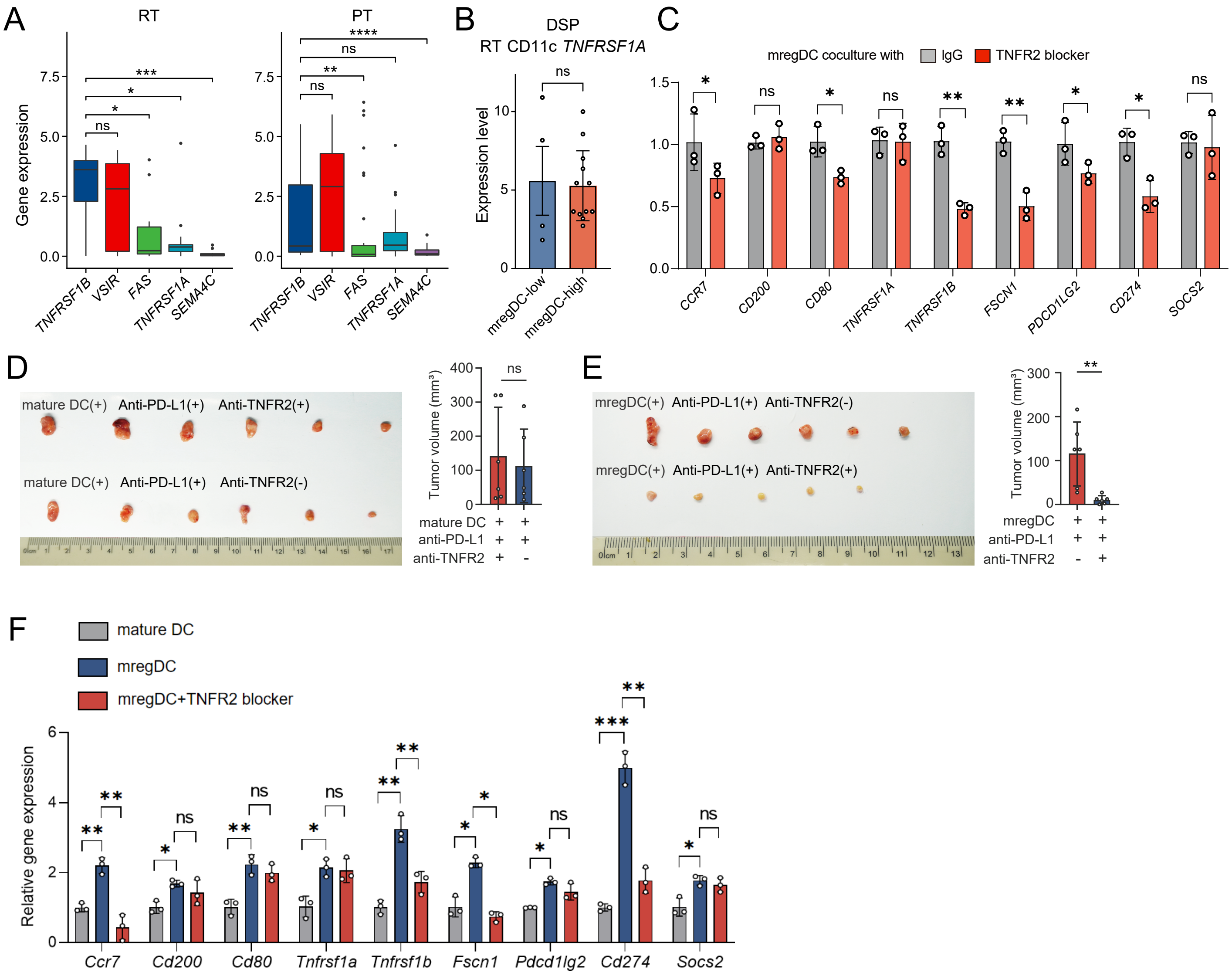

Supplementary figure 5. TNFR2 blockade can attenuate the mregDC program, related to Figure 5.

(A) Box plots comparing TNF interacting receptor *TNFRSF1A*, *TNFRSF1B*, *VSIR*, *FAS*, *SEMA4C* expression between PT and RT.

(B) The relative expression levels of *TNFRSF1A* were determined by qRT-PCR in mregDC-low group and mregDC-high group (8 patients in no recurrence group, 4 patients in recurrence group).

(C) MregDCs were treated with IgG or TNFR2 blocker. The relative expression levels of the indicated genes were determined by qRT-PCR (n=3 per group).

(D-E) Images of murine subcutaneous tumors and quantitative evaluation of tumor volume from mice receiving mature DCs (D) or mregDCs (E), treated with anti-PD-L1 antibody alone, or anti-PD-L1 antibody combined with anti-TNFR2 antibody (n=6 per group).

(F) The relative expression levels of the indicated genes in tumor-infiltrating mregDCs from mice with different treatments were determined by qRT-PCR (n=3 per group).

Supplementary Figure 6

A

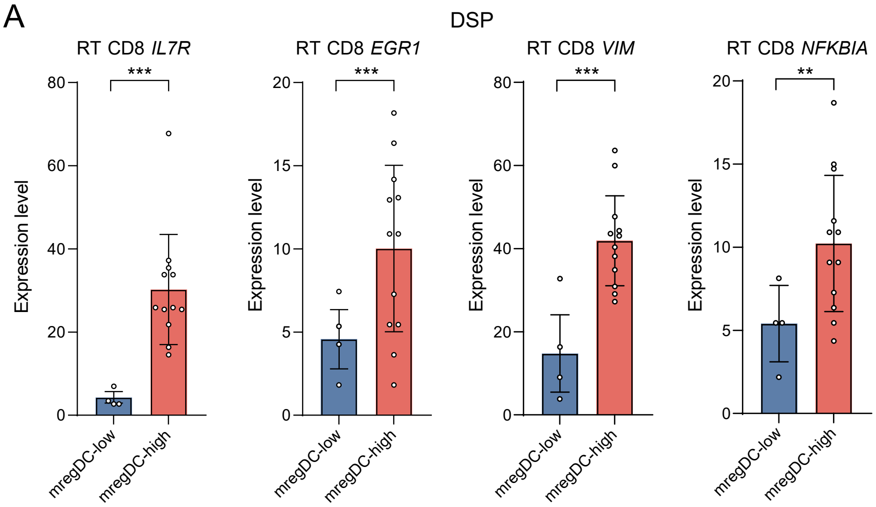

Supplementary figure 6. There is spatial co-localization between $CD161^+CD8^+$ T cells and mregDCs in early-relapse HCC, related to Figure 6.

(A) Bar plot showing genes characteristically highly expressed in $CD161^+CD8^+$ T cells were significantly increased in CD8 AOIs from mregDC-high group (8 patients in no recurrence group, 4 patients in recurrence group).

Supplementary Figure 7

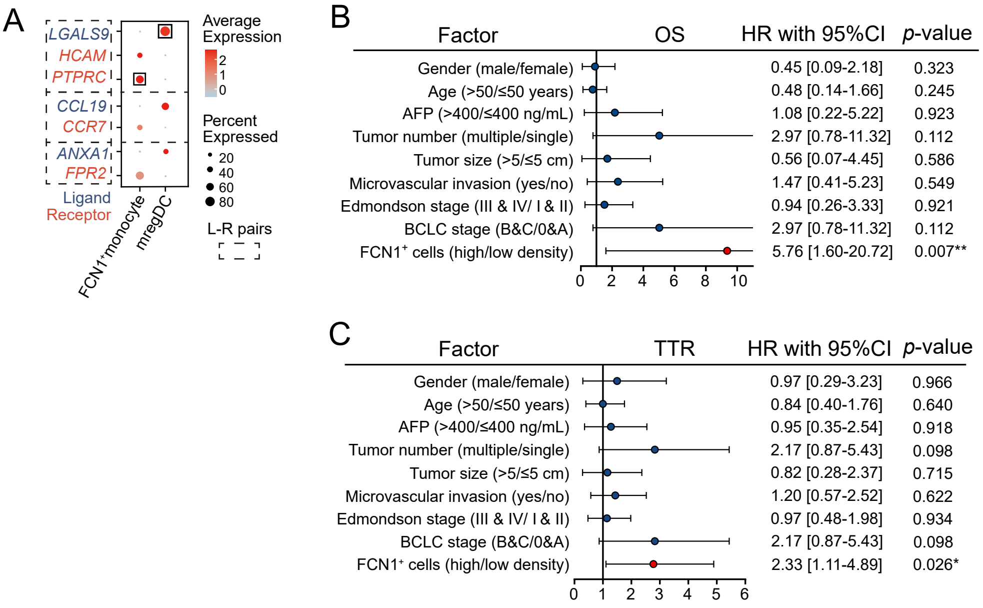

Supplementary figure 7. FCN1⁺ monocyte is an independent predictor for poor prognosis, related to Figure 7.

(A) CellChat analysis of intercellular crosstalk between mregDCs and FCN1⁺ monocytes using single-cell RNA-seq dataset.

(B-C) Cox proportional hazard ratio with 95% confidence interval for the density of FCN1⁺ monocytes, and multiple clinical parameters, regarding the overall survival (B) and time-to-recurrence (C). Statistical differences were tested using a Chi-square test.
